# Activation of the general stress response sigma factor SigB prevents competence development in *Bacillus subtilis*

**DOI:** 10.1128/mbio.02274-24

**Published:** 2024-10-29

**Authors:** Marco Harms, Stephan Michalik, Petra Hildebrandt, Marc Schaffer, Manuela Gesell Salazar, Ulf Gerth, Ulrike Mäder, Jan Maarten van Dijl, Michael Hecker, Uwe Völker, Alexander Reder

**Affiliations:** 1 University Medicine Greifswald, Center for Functional Genomics of Microbes, Interfaculty Institute for Genetics and Functional Genomics, Greifswald, Germany; 2 University of Greifswald, Center for Functional Genomics of Microbes, Institute of Microbiology, Greifswald, Germany; 3 Department of Medical Microbiology, University of Groningen, University Medical Center Groningen, Groningen, the Netherlands; Institut Pasteur, Paris, France

**Keywords:** competence development, ComK, general stress response, SigB, sporulation, Spo0A, as-*comK *(feature S365), ComK anti-sense RNA

## Abstract

Seemingly simple bacteria mount intricate adaptive responses when exposed to physical stress or nutrient limitation, and the activation of these responses is governed by complex signal transduction networks. Upon entry into the stationary growth phase, the soil bacterium *Bacillus subtilis* may develop natural competence, form biofilms or stress-resistant cells, or ultimately trigger a cellular differentiation program leading to spore formation. Master regulators, such as Spo0A, ComK, SinR, and SigB, constantly monitor the bacterium’s environment and then determine appropriate adaptive responses. Here, we show that exposure of *B. subtilis* to visible light and other stresses triggers a general stress response-dependent block in competence development. SigB serves as an “emergency system” to silence inappropriate expression of an alternative developmental program in the face of unfavorable conditions. In particular, we document a stress-dependent molecular mechanism that prevents accumulation of the central competence regulator ComK via expression of a SigB-driven antisense RNA (*as-comK*, S365) which is part of a noncontiguous operon.

**IMPORTANCE:**

*Bacillus subtilis* exhibits a large number of different specific and general adaptation reactions, which need to be well balanced to sustain survival under largely unfavorable conditions. Under specific conditions, natural competence develops, which enables *B. subtilis* to actively take up exogenous DNA to integrate it into its own genome. In contrast to this specific adaptation, the general stress response is induced by a variety of exogenous stress and starvation stimuli, providing comprehensive protection and enabling survival of vegetative *B. subtilis* cells. In the present work, we reveal the molecular basis for the interconnection of these two important responses in the regulatory network. We describe that the master regulator of the general stress response SigB is activated by physiological stress stimuli, including daylight and ethanol stress, leading to the inactivation of the competence master regulator ComK by transcriptional anti-sense regulation, showing a strict hierarchy of adaptational responses under severe stress.

## INTRODUCTION

**Fig 1:**
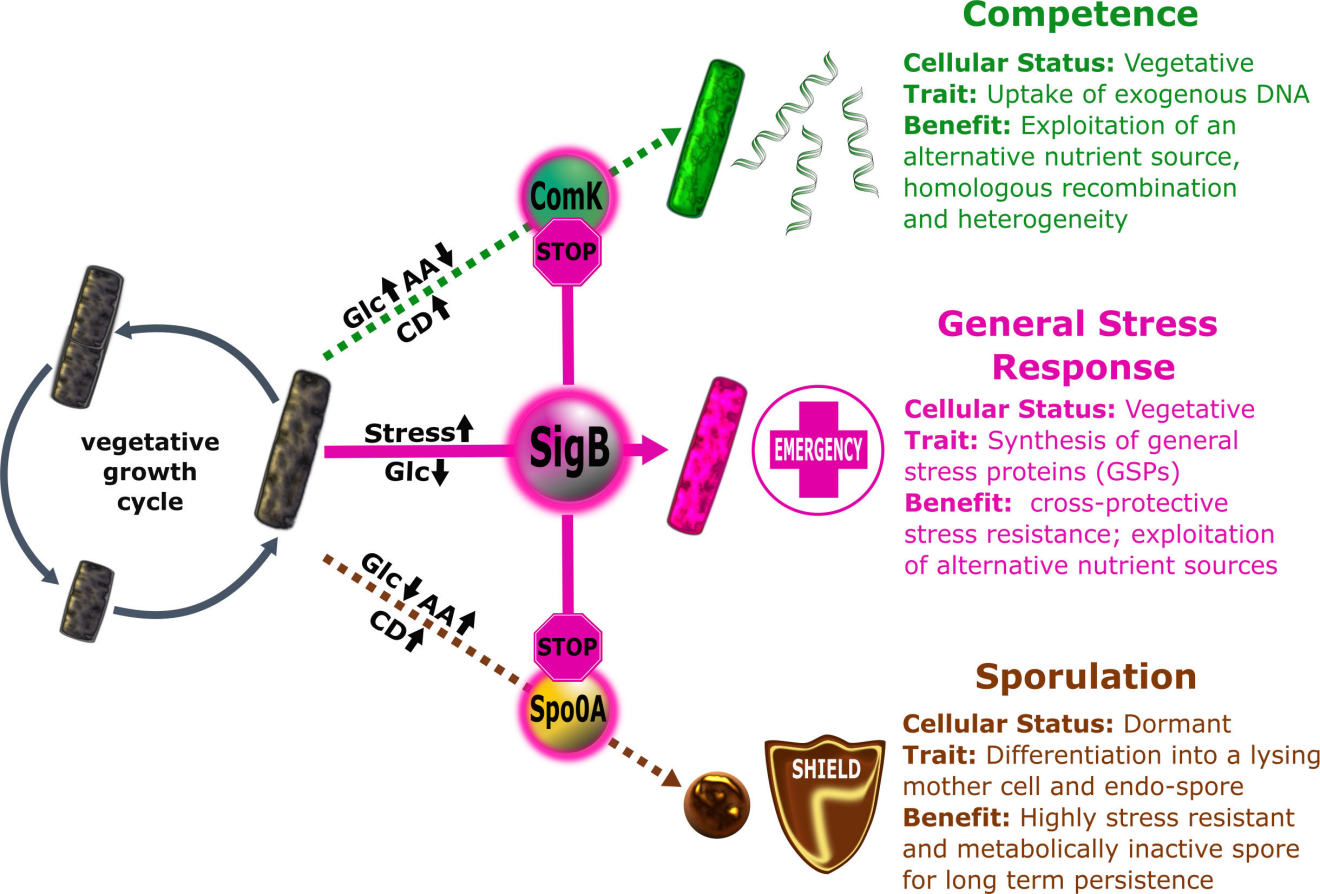
Simplified schematic overview of the growth cycle of *B. subtilis* and interactions between SigB and the master regulators ComK and Spo0A. Under conditions supporting growth, the vegetative cycle is favored (gray cycle). Natural competence (green path) is the ability of *B. subtilis* to bind and import extracellular DNA and is specifically induced under conditions of high cell densities (CDs) and limiting amino acid (AA) availability in the presence of glucose (Glc). Competence development is inherently coupled to the activity of the master regulator ComK. Sporulation (brown path) describes the development of a highly stress resistant endospore. It is also induced under conditions of high cell densities (CDs) and predominantly activated by glucose starvation (Glc) in the presence of amino acids (AAs). Initiation of sporulation depends on the activity of the primary master regulator Spo0A. The general stress response (pink path) is under control of the alternative sigma factor SigB that can be activated by a variety of physical stress stimuli (salt, heat, light, etc.) at any growth stage as well as glucose starvation at the onset of stationary phase. Stress-dependent activation of SigB directly inactivates the ComK and Spo0A regulators causing a block of competence and spore development.

In nature, growth of microorganisms is restricted by various adverse conditions, such as physical stresses and nutrient starvation. In order to thrive under these unfavorable conditions, microorganisms must constantly monitor their environment, process these data with the help of a complex regulatory network, and finally respond by adapting accordingly. Within this network, interconnected master regulators organize topology by amplifying or attenuating signals to ensure an appropriate response for survival of the population. The identification and functional characterization of specific nodes connecting master regulators in a given network facilitate comprehension of integrated responses and help to predict or deduce universally valid decision rules. The prospects of achieving these ambitious goals are best for already well-studied model organisms, like *Escherichia coli* or *Bacillus subtilis*. The soil bacterium *B. subtilis* can adapt to environmental insults using a wide collection of options that include development of natural competence, formation of biofilms and fruiting bodies (1), employing chemotaxis (2), secreting degradative enzymes (3), antibiotics and toxins for resource exploitation (4), generation of stress-resistant dormant vegetative cells (5–8), or spore formation (9, 10). Three well-studied adaptive responses that occur upon entry into stationary phase in *B. subtilis* are (i) the cellular differentiation into highly resistant dormant spores that is aimed at long-term persistence (9, 10), (ii) the development of natural competence allowing active uptake of extracellular DNA (11) for chromosome repair and adaptive genetic heterogeneity (12), and (iii) the general stress response, providing growing and non-growing cells with multiple preventive and cross-protective stress resistances (8, 13, 14) (Fig. 1). It is well established that the activities of the master regulators of sporulation, Spo0A, and competence development, ComK, are strongly intertwined in a highly complex sporulation-competence network—causing a strict physiological separation and temporal order of both responses (15, 16) (Fig. 1). Furthermore, the master regulator of the general stress response SigB does not only protect vegetative *B. subtilis* cells against acute life-threatening stress, but also orchestrates stress response and differentiation processes, such as sporulation (17–19). Physical stress stimuli mediate a SigB-dependent induction of the phosphatase Spo0E, that specifically targets and inactivates the master regulator of sporulation Spo0A by dephosphorylation causing a SigB-dependent block of spore development (17, 18) (Fig. 1). In *Listeria monocytogenes* particularly, blue light activation of SigB inhibits swimming motility, a crucial behavior for bacteria (20). The environmental cues trigger a shift in the bacterial behavior, redirecting their energy towards survival as well as pathogenicity and suppressing alternative energy-consuming differentiation programs.

Based on these observations, we previously proposed a model in which SigB performs two functions, (i) silencing the competing adaptive responses and (ii) simultaneously facilitating sufficient production of the general stress proteins that provide protection under acute physical stress conditions. In our present work, we show that the exposure to visible light and ethanol stress trigger SigB-dependent expression of a specific antisense transcript (*as-comK*) targeting *comK* mRNA. Expression of this antisense RNA reduces *comK* expression and ComK activity. Thereby, SigB directly inactivates competence development under SigB-activating stress conditions. This observation integrates the general stress response, competence development, and sporulation in the regulatory network of *B. subtilis* (Fig. 1). It also represents the first molecular dissection of a light-induced phenotype in *B. subtilis* and strongly supports the model of SigB as an “emergency system” that shuts off alternative developmental programs under acute physical stress conditions.

## RESULTS

### Stress-dependent SigB activation impairs transformability

**Fig 2:**
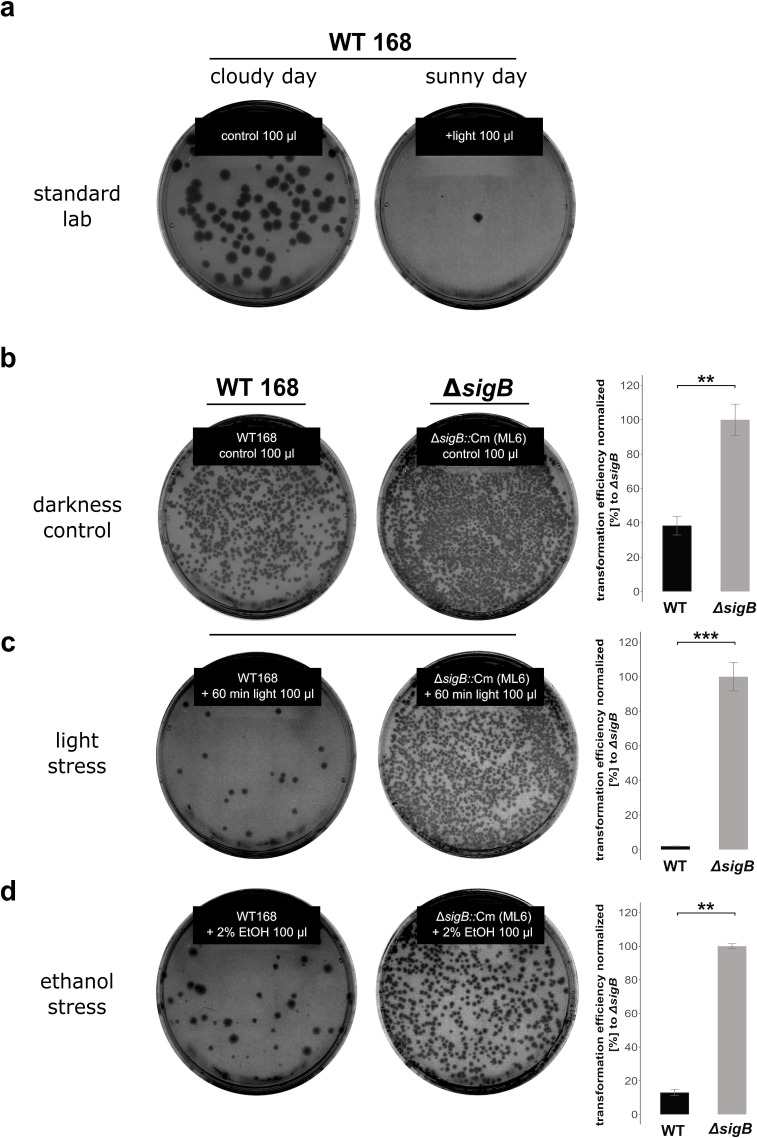
Stress-dependent reduction of transformation frequencies in *B. subtilis.* To determine transformation efficiencies, dilution series were plated onto both LB agar plates and selective LB agar plates to count the total colony forming units (CFU) and the transformed colony forming units (TCFU) after overnight incubation (24 h) at 37°C. Transformation frequencies (in percent) were determined by normalization on basis of the viable counts (CFU). (a) Two representative selective agar plates of replicate genetic transformation experiments of the *B. subtilis* wild-type 168 under standard laboratory conditions. The first experiment (left) was performed on a cloudy day and the second (right) on a bright sunny day. (b) Transformation experiments of the *B. subtilis* wild-type 168 and the isogenic Δ*sigB* mutant (ML6) under control conditions (darkness) and (c) after exposure to light (True Light LED source for 60 min) and (d) after 2% ethanol stress. The stress stimuli were applied when the cultures reached the late-exponential growth phase (OD_500_ = 1.6). Quantitative data from at least three independently performed biological replicates of the transformation assays are given as bar plots with the respective standard deviations. The transformation frequencies (Table S4) for the control, light, and ethanol stress experiments were normalized to those of the Δ*sigB* mutant. Statistical significance within the bar plots is represented by asterisks (**, *P*-values ≤ 0.01; ***, *P*-values ≤ 0.001).

It is a common observation that transformation efficiency can vary substantially in *B. subtilis*. Critical evaluation of standard laboratory conditions revealed that the exposition of growing cultures to daylight has an inhibitory effect on competence development in *B. subtilis* that has been neglected so far. Figure 2a illustrates the outcome of two identical transformation experiments that were performed on two consecutive days with the *B. subtilis* wild-type strain 168 in a laboratory with daylight exposure in summer (June). The only difference between both experiments was that the first transformation experiment (Fig. 2a, left) was performed on a cloudy day and the second (Fig. 2a, right) on a bright sunny day, suggesting that exposure to different light intensities apparently influenced transformation efficiencies. To support this idea, the experiment was repeated in complete darkness (Fig. 2b, left). This simple change in cultivation conditions drastically increased transformation efficiency of the wild-type 168 compared with two “standard laboratory conditions” shown in Fig. 2a. Since it is well known that the activity of the general stress sigma factor SigB is also stimulated by radiation with visible light (21–24), we examined the role of SigB for competence development. Thus, transformation efficiency was comparatively profiled in the *B. subtilis* wild-type 168 and its isogenic Δ*sigB* mutant. Interestingly, the transformation frequencies observed for the Δ*sigB* mutant were already 2.5-fold higher during incubation in complete darkness compared with those of the wild-type strain, indicating that very likely, even basal level activity of SigB in the wild-type has a negative effect on competence development (Fig. 2b; Table S4). Irradiation of the cultures with an artificial full-spectrum daylight source for 60 min during exponential growth caused a dramatic reduction in transformation efficiency of the wild-type by 98% (50-fold) but not in the isogenic Δ*sigB* mutant (Fig. 2c; Table S4). To further substantiate this apparent involvement of SigB, we also used 2% ethanol stress as another typical SigB-activating stimulus, and again significant reduction of transformation efficiency by 92% was observed in the wild-type strain (Fig. 2d; Table S4).

### SigB induction independent of stress suppresses competence development in *B. subtilis*

**Fig 3:**
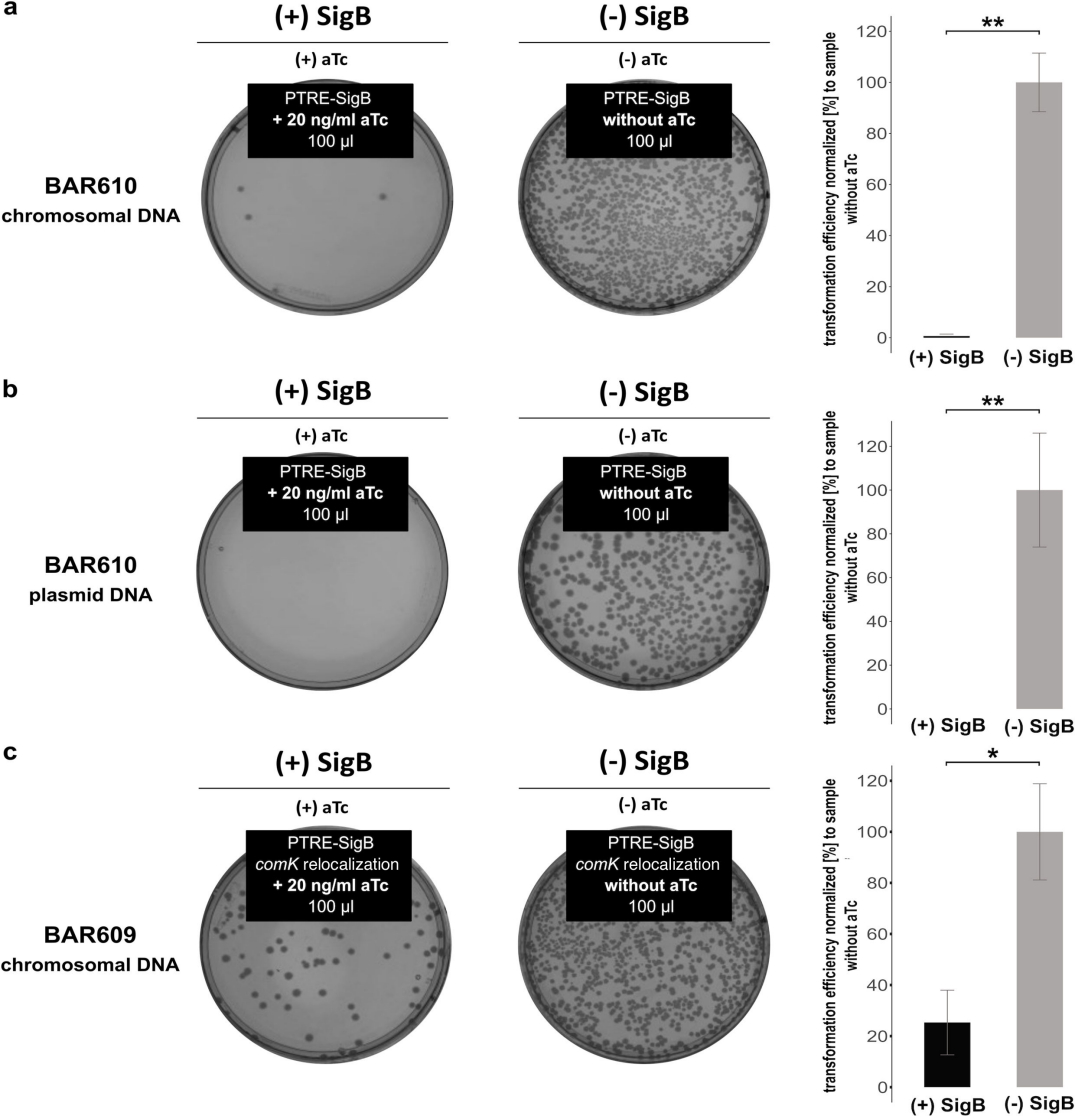
SigB-dependent reduction of transformation frequencies in *B. subtilis.* To determine transformation efficiencies, dilution series were plated onto both LB agar plates and selective LB agar plates to count the total colony forming units (CFU) and the transformed colony forming units (TCFU) after overnight incubation (24 h) at 37°C. Transformation frequencies (in percent) were determined by normalization on the basis of the viable counts (CFU). Transformation of (a) chromosomal DNA and (b) plasmid DNA: Two representative selective agar plates of replicate genetic transformation experiments of the strain BAR610 carrying an inducible copy of the *sigB* gene under strict transcriptional control of a SigA-type promoter repressed by the tetracycline repressor TetR (26) are shown. SigB was either induced by the addition of 20 ng/mL anhydrotetracycline [aTc; left panels, (+)SigB] or remained uninduced [right panels, (−)SigB]. Transformation of (c) chromosomal DNA in strain BAR609: this strain has decoupled *comK* expression from the native chromosomal locus and represents a *comK* complementation mutant in the BAR610 background. A detailed description of the strain is given later in the text but is included here for direct comparison with BAR610. Two representative selective agar plates of replicate genetic transformation of the strain BAR609 with chromosomal DNA are shown. Quantitative data from at least three independently performed biological replicates of the transformation assays are represented by bar plots with the respective standard deviations. The transformation frequencies were normalized to those of the uninduced control cultures, respectively. Statistical significance within the bar plots is represented by asterisks (*, *P*-values ≤ 0.05; **, *P*-values ≤ 0.01).

In order to distinguish the direct effect of SigB from potential pleiotropic effects caused by different stress stimuli, an inducible system for SigB expression was generated in the original chromosomal locus of the *sigB*-operon (strain BAR610). Here, the SigB-type promoter of the tetra-cistronic operon *rsbV-rsbW-sigB-rsbX* was replaced by an anhydrotetracycline-inducible SigA-type promoter construct (P_TRE_ - promoter with tetracycline responsive elements). Simultaneously, the genes *rsbV-rsbW-rsbX* were deleted to abolish control of SigB activity by partner switching (25) (Fig. S2). Due to the strict repression of P_TRE_ by the tetracycline repressor TetR (26), the basal level expression of SigB is very low under control conditions and induced by the addition of anhydrotetracycline (aTc), resulting in the production of active SigB in the absence of additional stress stimuli. In agreement with the expectation, competence development and transformability were completely abolished in the conditional P_TRE_-*sigB* strain (BAR610) upon addition of aTc (Fig. 3a), which could be caused by two different effects/events. On the one hand, SigB could already suppress the uptake of extracellular DNA or only suppress the homologous recombination of newly acquired DNA intracellularly. In order to distinguish between these two possibilities, we performed a control experiment with plasmid DNA (Fig. 3b) in addition to the transformation experiment with chromosomal DNA (Fig. 3a). In fact, active SigB also suppressed the transformability of cells with plasmid DNA, so that already the DNA uptake into the cell is very likely affected and not the recombination event.

### SigB causes expression of the *comK* antisense RNA *as-comK* (S365)

**Fig 4:**
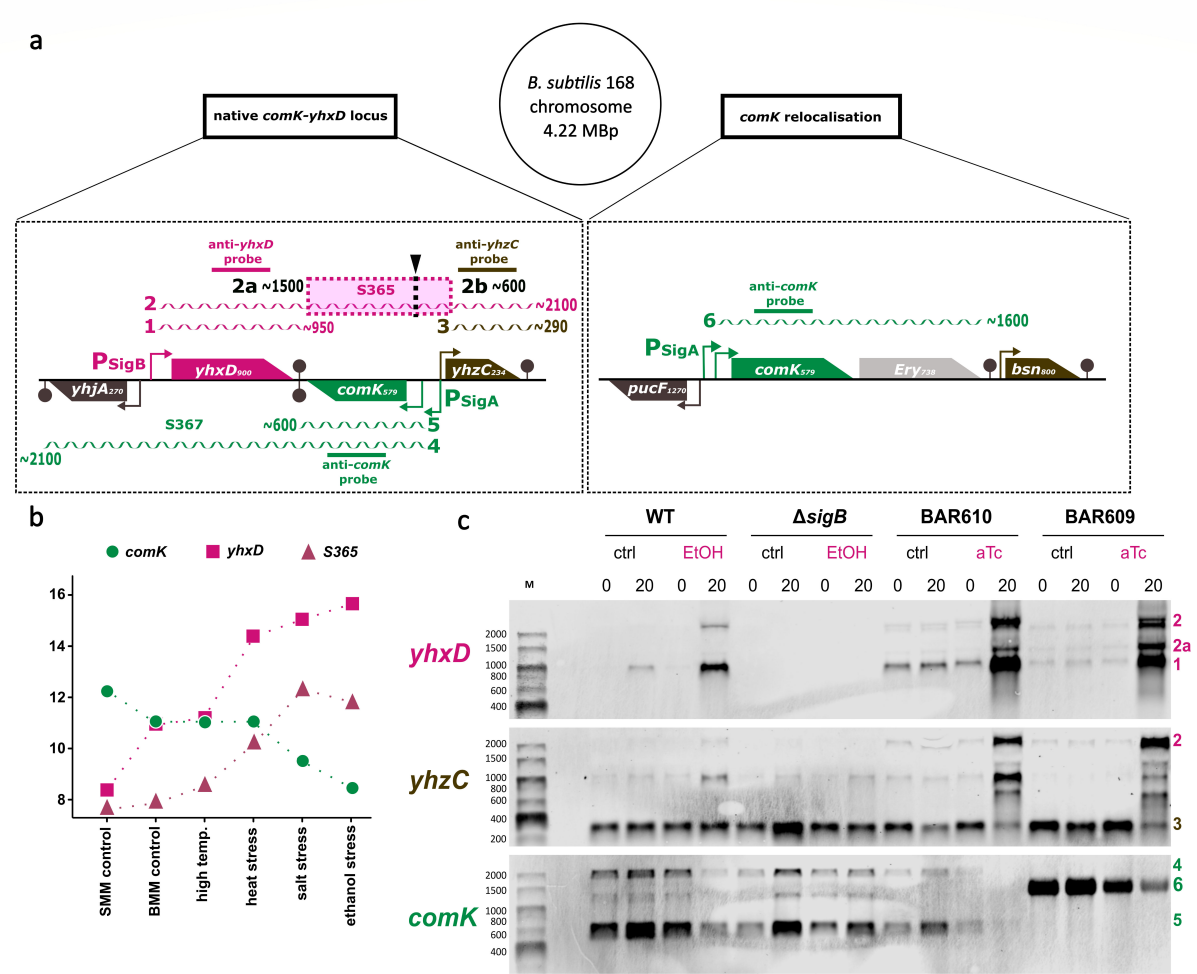
Analysis of the impact of SigB activity on transcript patterns in the *yhxD-comK* region of the *B. subtilis* chromosome. (a) Schematic representation of the chromosomal *yhxD-comK* region. Genes are depicted (*yhjA*, *yhxD*, *comK*, and *yhzC*) together with their gene length in bases (small number adjacent to gene names), promoters (colored arrows), terminators (dark grey paddles), detected mRNAs (numbered consecutively from 1 to 6 and colored according to their promoter(s) of origin as well as the three RNA probes directed against the *yhxD*, *yhzC*, and *comK* transcripts used for the detection of all possible transcripts in this region (colored according to the transcript to be detected). Transcript number 2 includes three parts, the *yhxD* coding region (5′ end), the *comK* antisense as-*comK* (S365) region (middle) as well as the terminator readthrough into the coding region for *yhzC* (3′ end) detected by either the *yhxD* probe or the *yhzC* probe. Transcript 2 is most likely processed post-transcriptionally and split into parts 2a (detected by the *yhxD* probe) and 2b (detected by the *yhzC* probe). The putative cleavage position in transcript 2 is marked by a black triangle and a dotted line. Transcripts 4 and 5 both include the native *comK* coding region. While transcript 5 represents the monocistronic *comK* terminated at the terminator at the 3’ end of *comK*, transcript 4 represents the long read through *comK-S367* transcript (both detected by the *comK*-probe). Transcript 6 corresponds to the *comK* mRNA originating from the translocated *comK* gene locus (BAR609). (b) Inversely correlated expression profiles of *yhxD* (pink), as-*comK* (S365) (dark purple) and *comK* (green) mRNAs [adapted from (27)]. The *yhxD*, as-*comK* (S365), and *comK* expression levels are displayed for selected growth and stress conditions. Culture conditions from Nicolas et al. (27) were ordered according to increasing *yhxD and* S365 expression levels (SMM control: cells sampled at OD_500_ 0.4 from cultures of the BSB1 wild-type strain in Spizizens minimal medium (SMM) at 37°C with vigorous shaking, BMM control: cells sampled at OD_500_ 0.4 from cultures of the BSB1 wild-type strain in Belitsky minimal medium (BMM) at 37°C with vigorous shaking, high temperature: cells sampled at OD_578_ 1.0 from cultures of the BSB1 wild-type strain continuously grown in SMM at 51°C with vigorous shaking, heat stress: cells sampled at approximately OD_500_ 0.4 from cultures of the BSB1 wild-type strain in BMM at 48°C for 10 min with vigorous shaking, salt stress: cells sampled at approximately OD_500_ 0.4 from cultures of the BSB1 wild-type strain in SMM with 0.4 M sodium chloride for 10 min with vigorous shaking, ethanol stress: cells sampled at approximately OD_500_ 0.4 from cultures of the BSB1 wild-type strain in BMM with 4% ethanol for 10 min with vigorous shaking). (c) Northern blot experiments displaying the expression profiles of the *yhxD*, *as-comK* (S365), *yhzC,* and *comK* transcript under control conditions (ctrl; promoting *comK* expression) and 20 min (T20) after addition of ethanol (WT and Δ*sigB*) or anhydrotetracycline (aTc)-dependent induction (BAR610 and BAR609). Cells were grown in competence medium in the dark and 2% ethanol or 20 ng/mL anhydrotetracycline was added when cultures reached an optical density of OD_500_ = 1.6. The respective detection probes used are shown on the left highlighted in their respective color (*yhxD*, *yhzC,* and *comK*) and their relative localizations are given in panel a. Detected transcripts are numbered and color-coded according to the schematic presentation in panel a. Putative cleavage products of transcript 2 are labeled with 2a.

Having shown that the activity of the alternative sigma factor SigB is responsible for a block of competence development and transformability, we sought to identify the molecular mechanism or regulatory link interconnecting both systems. A very promising candidate for a direct SigB-dependent prevention of the production of the competence master regulator ComK emerged from the analysis of a previous comprehensive transcriptome study of *B. subtilis* by Nicolas and coworkers (27). This study had shown that the *comK* gene is divergently transcribed in a tail-to-tail orientation with the gene *yhxD* (Fig. 4a left), which is strongly induced in a SigB-dependent fashion (28, 29). In particular, the previous transcriptome study (27) indicated the production of a potential *comK* antisense RNA named S365 (Fig. 4a pink dotted box), which originates from the imperfect termination of transcription of mRNA starting at the SigB promoter in front of *yhxD*. Such natural antisense RNAs, which overlap other transcriptional units, are common in bacteria (30). Furthermore, a comparison of the *yhxD* and *comK* transcription profiles of this previous study (27) also indicated that the expression of both genes is inversely correlated (Fig. 4b). Under conditions where expression of *yhxD* and S365 (*as-comK*) (Fig. 4b pink graphs) is high, transcription of *comK* is low (Fig. 4b green graph), and *vice versa*. To verify these data in our experimental setup, we performed a Northern blot experiment (Fig. 4c) with total RNA isolated from *B. subtilis* wild type, the isogenic Δ*sigB* mutant, as well as the BAR610 strain carrying the anhydrotetracycline-inducible *sigB* copy. We started this study using light as the stimulus inducing *sigB* expression and subsequently increased SigB activity. Specifically, Northern blot analysis was used to proof SigB-dependent light inducibility of the *yhxD-as-comK* expression (Fig. S5) and the corresponding decline in ComK activity was shown in FACS experiments with a strain carrying a *comG*-GFP reporter gene fusion (Fig. S6). Since light stress as a SigB-inducing stimulus was more difficult to handle, the well-established ethanol stress stimulus for SigB activation was chosen for the detailed mechanistic analysis. From control cultures grown in competence medium in the absence of ethanol or aTc, one sample was taken in the late exponential growth phase (OD_500_ = 1.6 t0 min) and a second sample 20 min later in the transient phase (Fig. S1). *comK* expression was observed in both samples (Fig. 4c, transcripts 4 and 5). For the ethanol stress and stress free aTc-mediated induction of *sigB,* an identical control sample was harvested (OD_500_ = 1.6 t0 min) right before addition of 2% ethanol for the wild-type and Δ*sigB* culture and before aTc addition to the BAR610 culture. The corresponding ethanol-/aTc-treated samples were also taken 20 min after ethanol or aTc addition in the transient phase (Fig. S1). Three RNA probes (for *yhxD,* for *comK* and for *yhzC*) were used to detect all transcripts originating from the *yhxD-comK* region (Fig. 4a and c). Indeed, all transcripts (labeled 1 to 6) presumed in the previous Tiling array study (27) could be identified by our Northern blot analysis (Fig. 4c). Importantly, we were able to verify the presence of the SigB-dependent induction of the *comK* antisense transcript *as-comK* as the central part of transcript 2 originating from the *yhxD* SigB-type promoter (Fig. 4a and c). This transcript comprises the genes *yhxD* and *yhzC* as well the intergenic region between them with *comK* encoded on the opposite strand. Thus, this constellation constitutes a non-contiguous operon as defined by S. Sáenz-Lahoya and coworkers (31). SigB dependence of the *yhxD* transcripts (Fig. 4c—transcripts 1 and 2) is clearly demonstrated by their absence in the Δ*sigB* mutant. A weak signal for the monocistronic *yhxD* transcript (transcript 1—approx. 950 b) can be detected under control conditions in the wild-type t20 samples due to natural transient phase activation of SigB triggered by nutrient exhaustion (32). Upon ethanol stress, two distinct transcripts originate from the *yhxD* promoter. A strong signal for the monocistronic *yhxD* transcript (transcript 1—approx. 950 b), terminated at the bi-directional terminator in between *yhxD* and *comK* and a second signal for the *yhxD-S365-yhzC* read-through transcript (transcript 2—approx. 2,100 b). The latter transcript (transcript 2) can also be detected with the *yhzC* probe together with the monocistronic *yhzC* transcript (transcript 3—approx. 290 b) originating from its own *yhzC* promoter. An increase of *comK* transcript from late exponential (t0 min) to transient phase (t20 min) (Fig. 4c—transcripts 4, 5, and 6) could be observed for all control samples from all strains tested due to the chosen growth conditions and time points promoting competence development and positive ComK autoregulation. As expected, the ethanol stress induction of transcript 2 containing the *comK* antisense region *as-comK* leads to a simultaneous reduction of the *comK* mRNAs in the *B. subtilis* wild type compared with the respective control samples (t0 min). Furthermore, the Δ*sigB* strain displays a reduced but clear induction of the *comK* transcript after addition of ethanol stress in comparison to its t0 min control samples.

With regard to the stress-free induction of active SigB in the BAR610 strain, expression of *yhxD* transcripts 1 and 2 was already evident under control conditions, indicating that some SigB activity is already observed in this strain under control conditions. In agreement with this statement, the amounts and induction strength of *comK* transcript were strongly decreased in the control samples of strain BAR610 (Fig. 4c—transcripts 4 and 5) compared with the wild-type controls. Furthermore, a strong induction of transcripts 1 and 2 from the *yhxD* SigB-type promoter was observed in the BAR610 strain after aTc-dependent SigB expression, which simultaneously led to a complete elimination of the *comK* transcripts (Fig. 4c). Taken together, these findings strongly indicate that activation of SigB prevents expression of competence genes by mediating production of the *comK* antisense RNA as-*comK* (S365) that prevents production of sufficient levels of ComK for competence development.

In order to exclude a possible effect of the co-transcribed gene *yhxD* on competence development, a translation null mutant of *yhxD* was created by deleting the ATG start codon of *yhxD*. This translational null mutant of *yhxD* alone behaved identical to the wild type. Thus, the SigB-dependent expression of the YhxD protein has no effect on competence development (data not shown).

### Induction of *as-comK* (S365) alone blocks competence development

**Fig 5:**
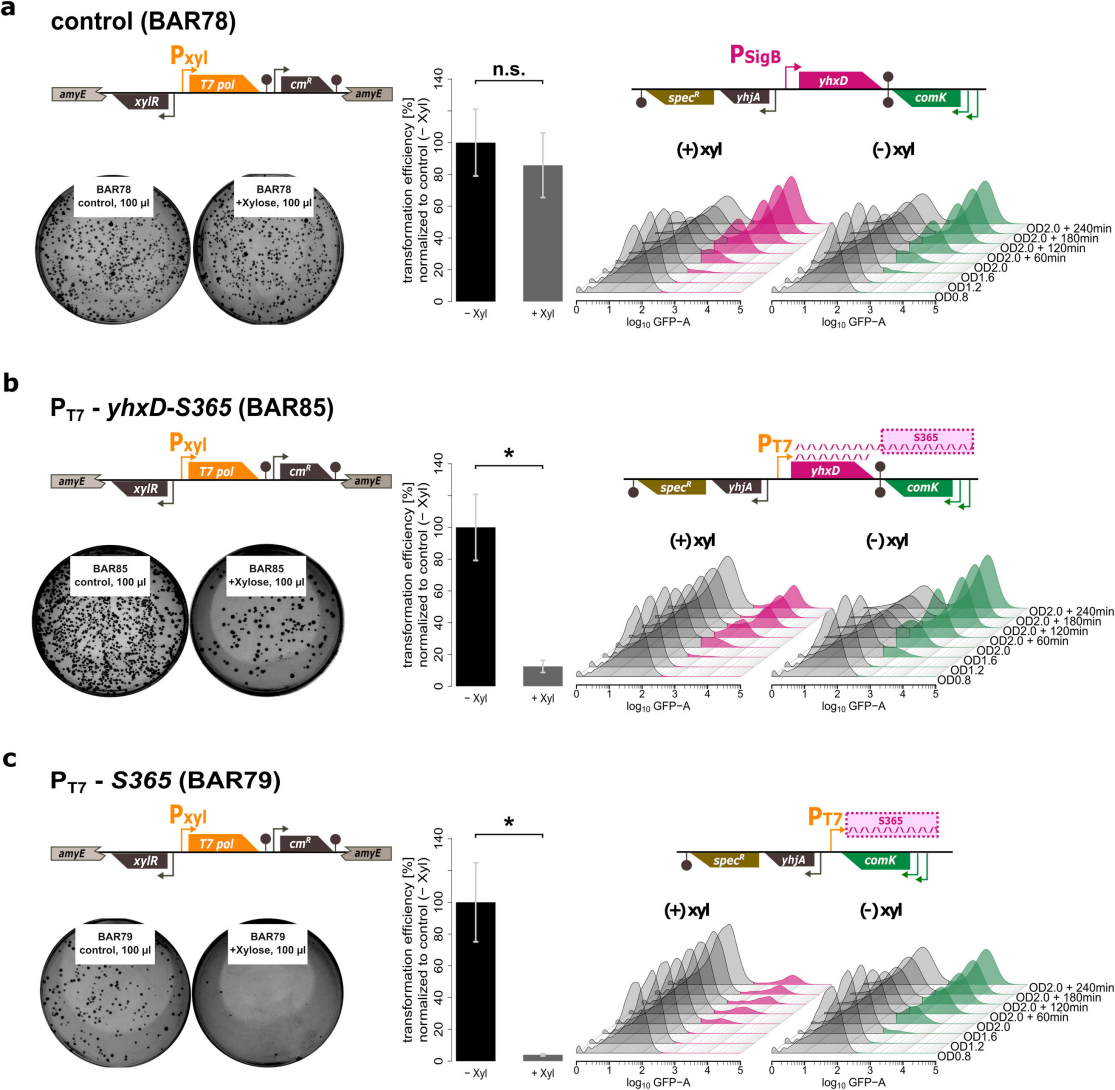
Induction of the antisense transcript *as-comK* (S365) inhibits competence development. Schematic representation of the chromosomal organization of the tested mutant strains (BAR78, BAR85, and BAR79) and display of the results of transformation assays (left column) and FACS measurements of a *comG-gfp* reporter gene fusion (right column). All three strains tested carry a xylose-inducible copy of the T7 polymerase gene (T7 *pol*) that is chromosomally integrated into *amyE* and a P*_comG_-gfp* reporter gene fusion in *comG*. Cells were either grown without (−xyl/green—right panel) or with (+xyl/pink—left panel) xylose. Left column: two representative selective agar plates of replicate genetic transformation experiments as well as bar charts of the determined transformation frequencies (normalized to the non-induced control cultures) are shown. Right column: histograms of the FACS population dynamic measurements are shown, in which the non-competent subpopulations are depicted in gray, and the competent subpopulations are either colored in green (non-induced controls) or purple (induced T7 polymerase), respectively. (a) No modifications were introduced into the *yhxD-comK* region in the control strain (BAR78). Therefore, expression of the T7 polymerase does not affect *yhxD-as-comK*(S365) induction. (b) In the P_T7_-*yhxD-as-comK*(S365) strain (BAR85), the SigB-dependent promoter (P_SigB_) in front of *yhxD* has been replaced by a T7 promoter (P_T7_) allowing induction of the *yhxD* and *yhxD-as-*comK(S365) transcripts by xylose. (c) In the P_T7_-*as-comK*(S365) strain (BAR79), the entire region containing the SigB-dependent promoter (P_SigB_), the *yhxD* coding sequence and the bidirectional terminator were deleted, and a T7 promoter (P_T7_) was integrated immediately upstream of *as-comK* (S365), allowing induction of the *as-comK* transcript by xylose alone. Statistical significance within the bar plots is represented by asterisks (*, *P*-values ≤ 0.05).

Next, we wanted to test the hypothesis that the induction of the antisense transcript *as-comK* (S365) is responsible for the SigB-dependent block of competence development. This set of experiments was designed to assess the impact of the antisense transcript on both transformation efficiency and the activity of ComK. The activity of ComK was monitored *in vivo* with the help of a P*_comG_-gfp* reporter gene fusion, the expression of which directly depends on ComK activity. To accomplish these goals, three additional mutant strains were constructed. The first strain (BAR78) served as a control and contained a xylose-inducible copy of the T7-polymerase gene (T7 *pol*) that was chromosomally integrated into the *amyE* locus as well as the P*_comG_-gfp* reporter gene fusion as described by Smits et al. (33), but exhibited no modifications in the *yhxD and comK* region (Fig. 5a). In contrast, the next two strains allowed a xylose-mediated induction of either the *yhxD* and *yhxD-as-comK* transcripts (P_T7_-*yhxD-as-comK*; BAR85) (Fig. 5b) or the *as-comK* transcript alone (P_T7_-*as-comK*; BAR79) (Fig. 5c) from an inserted T7-polymerase promoter (P_T7_). All three strains were either grown without (−xyl) or with (+xyl) expression of the T7 polymerase and transformation assays as well as monitoring of the expression of the ComK-dependent *comG-gfp* reporter gene fusion by FACS were performed. For the control strain neither the transformation frequencies nor the expression of the P*_comG_-gfp* reporter gene fusion showed any significant differences between the induced and non-induced cultures (Fig. 5a). Instead, induction of the *yhxD* and *yhxD-as-comK* transcripts from the upstream P_T7_ promoter (strain BAR85) (Fig. 5b) resulted in both, a decrease of transformation frequencies to 13% (7.7-fold decrease), as well as a decrease of the GFP-expressing competent population to 24% (2.75-fold decrease) compared to the 66% GFP-positive population reached in the non-induced control cultures (Fig. 5b). Finally, induction of the *as-comK* transcript alone (Fig. 5c) resulted in an even more drastic drop of transformation frequencies to only 4% (25-fold decrease) as well as a decrease of the GFP-expressing population to 11% (3.8-fold) compared with 42% reached in the non-induced control cultures (Fig. 5c). Thus, expression of the *as-comK* transcript alone in the absence of any stress stimulus triggered reduced transformation efficiency and reduced ComK activity. Based on the result that a SigB null mutant is hypercompetent (Fig. 2b), we wanted to test whether silencing of *yhxD-as-comK* transcription alone also leads to hyperactivation of ComK and competence gene expression. To show this, we generated a strain (BAR604) carrying a SigB promoter null mutation abolishing *yhxD-as-comK* transcription (see Fig. S7a). Indeed, decoupling the expression of *yhxD-as-comK* from SigB activity alone also causes significantly higher transformation frequencies compared with the wild type (see Fig. S7b).

Expression of antisense RNAs might directly reduce expression of genes of the opposite strand by interfering with the transcriptional machinery or impact expression of target genes via binding to target RNAs and then reducing translation by inhibiting ribosome binding and/or decreasing mRNA stability. In order to distinguish between these two options, a *comK* complementation strain was created. In strain BAR609 *comK* expression was uncoupled from the native chromosomal regulation but the transcript originating from the SigB-type *yhxD* promoter also representing the untranslated but fully transcribed *yhxD-as-comK* mRNA was not modified. In this strain, also the promoters of *comK* were deleted at the native chromosomal locus to prevent expression of *comK* from this locus, leaving the coding sequences of the *comK* and the surrounding native chromosomal context intact. To complement the silenced *comK* gene in the native locus, the *comK* gene was integrated into another chromosomal location along with its original regulatory regions, allowing native *comK* expression just from another chromosomal site (detailed in Materials and Methods and Fig. S4). Furthermore, the stress-free induction system for SigB from BAR610 was cloned into this strain, allowing stress-free induction of SigB in the complementation background of *comK* by addition of aTc. If the SigB effect was mediated by interfering directly with transcription of *comK* in the direction opposite to *as-comK*, transformability in BAR609 would be expected to be independent of SigB activity because of *comK* transcription and *as-comK* transcription had been physically separated. However, the transformation frequency of BAR609 was still significantly affected by the production of active SigB after addition of aTc in strain BAR609 (Fig. 3c). A direct comparison with strain BAR610 (Fig. 3c) revealed that after *comK* relocalization, the SigB effect was reduced, but this might easily be explained by the lower probability of pairing between *as-comK* RNA and *comK* mRNA if both genes are not located at the same locus but in *trans* (see Fig. 4a). Since transformability is still significantly influenced by SigB activity, the effect of *as-comK* is likely mediated by binding to *comK* mRNA no matter whether it is produced in *cis* or *trans* and then reducing translation by inhibiting ribosome binding and/or decreasing mRNA stability. Measurements of the mRNA levels of *comK* and *yhxD-as-comK* in BAR609 support this notion and even indicate that *as-comK* expression directly triggered *comK* mRNA destabilization (Fig. 4c). BAR609 displayed high levels of *comK* mRNA under control conditions (no aTc addition and minor SigB activity), and these levels were significantly decreased after aTc addition and concomitant production of active SigB (Fig. 4c). This decrease in *comK* mRNA levels was likely caused by the strong increase of *as-comK* produced in *trans* (Fig. 4c).

### Determination of SigB levels and activity

If SigB activities reach non-physiologically high levels, they become detrimental to growth and cause cell lysis or strong pleiotropic and unwanted regulatory effects (5, 25, 34). To ascertain that stress-free induction of SigB triggered SigB activities in the physiological range, levels of SigB and SigB activity were monitored by a combination of Western blot analysis and comprehensive global proteome profiling. For this purpose, the *B. subtilis* wild-type 168, an isogenic Δ*sigB* mutant and the two strains BAR610 and BAR609 were analyzed under control and inducing conditions.

**Fig 6:**
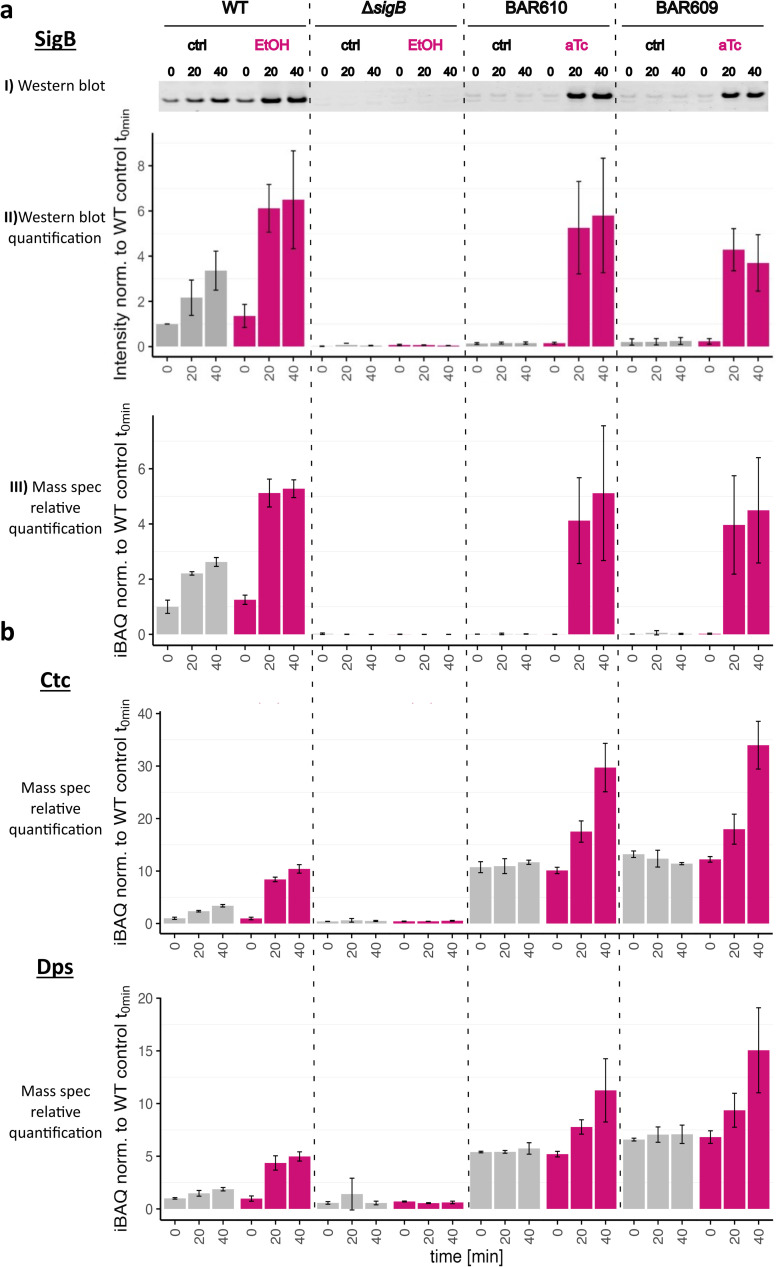
Comparative analysis of SigB protein quantity and SigB activity in the strains *B. subtilis* wild-type 168, Δ*sigB,* and BAR610 and BAR609. (a) Monitoring of SigB amount: Sections of a representative Western blot against SigB protein detected by NIR fluorescence measurement are displayed. Below the corresponding bar plots and standard deviations of the quantifications of three Western blots from independent biological replicates are shown. The respective values were normalized against the wild-type sample at control time t0. Furthermore, bar plots of the iBAQ intensities of SigB with standard deviations from a data independent acquisition–mass spectrometry (DIA-MS) analysis are shown. Three independent biological replicates were measured. The respective measured quantities were normalized against the wild-type sample at control time t0. (b) Analysis of SigB activity: Proteomic profiling allowed simultaneous analysis of the levels of SigB and two members of the SigB regulon, *ctc* and *dps*, and thus SigB activity. Bar plots of the iBAQ intensities of two SigB dependently expressed proteins, Ctc and Dps, with standard deviations from a DIA-MS analysis are displayed. Three independent biological replicates were measured. The respective quantities were normalized against the wild-type sample at control time t0.

The direct comparison of ethanol induction in the wild type and Δ*sigB* background and the stress-free SigB induction using aTc in the BAR610 and BAR609 strains allowed the evaluation of the physiological amounts and activity levels of SigB in all strains tested. Protein levels (Fig. 6a) and activity of SigB (Fig. 6b) were determined to directly compare the *B. subtilis 168* wild type and Δ*sigB* mutant as well as the stress-free anhydrotetracycline-inducible SigB constructs (BAR610 and BAR609). Samples were taken from control cultures grown in competence medium in the late exponential growth phase (OD_500_ = 1.6), and 20 min as well as 40 min after this time point (Fig. S1). For ethanol stress (2%, wild type and Δ*sigB*) and stress-free induction (20 ng/mL aTc, BAR610 and BAR609) samples were collected before, 20 min, and 40 min after the addition of the stimuli, respectively. SigB levels were evaluated by two independent approaches, near infrared (NIR) fluorescent Western blot as well as data independent acquisition–mass spectrometry (DIA-MS) (Fig. 6a). Both, exposure to ethanol stress and induction by aTc resulted in almost identical protein levels of SigB in all strains tested, except the negative control strain Δ*sigB*. To test the activity of SigB, protein levels of two well-known members of the SigB regulon, Ctc and Dps, were monitored by DIA-MS, and the data are shown in Fig. 6b. The observed activity level of SigB was found to be approximately threefold higher in the stress-free anhydrotetracycline-inducible systems BAR610 and BAR609 compared with the ethanol-stressed wild-type samples. This increase is easily explained by the uncoupling of SigB from its regulatory partner switch module RsbW-RsbV in strains BAR610 and BAR609 (Fig. 6b). Nevertheless, these data in combination with the monitored growth curves (Fig. S1) indicate that the amount of SigB as well as the SigB activity levels were not detrimental to growth.

### SigB activation affects ComK levels and competence proteins

**Fig 7:**
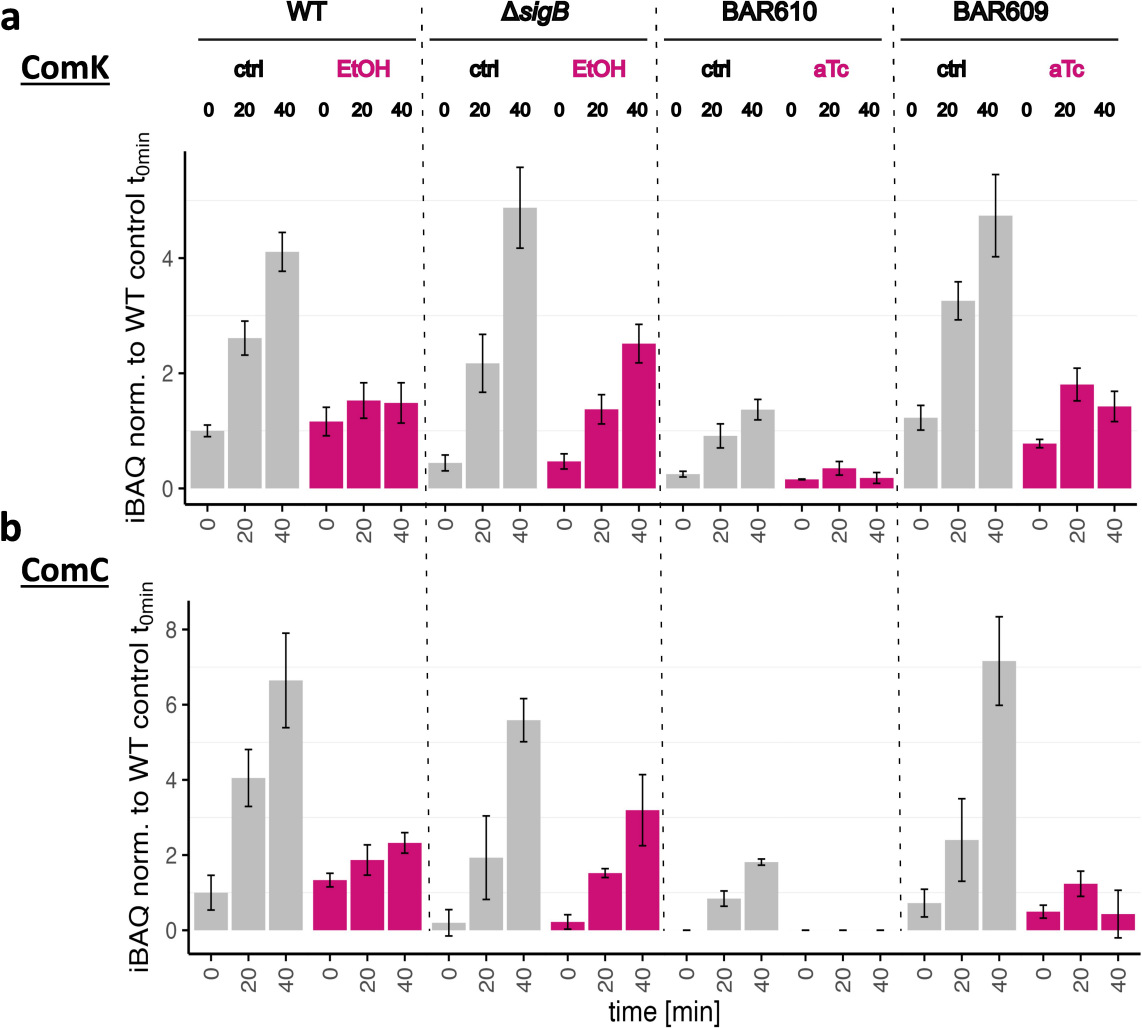
Comparative analysis of ComK protein quantity and ComK activity in the strains *B. subtilis* wild-type 168, Δ*sigB,* and BAR610 and BAR609. (a) Monitoring of ComK amount: bar plots of the iBAQ intensities of ComK with standard deviations from a DIA-MS analysis of three independent biological replicates are shown. The respective quantities were normalized to the wild-type control sample t0. (b) Analysis of ComK activity: bar plot of the iBAQ intensities of the ComK dependently expressed protein ComC with standard deviations from a DIA-MS analysis are displayed. Three independent biological replicates were measured. The respective measured quantities were normalized to the wild-type control sample t0. The data originate from the same proteome profiling experiment displayed in Fig. 6.

Since ComK controls the expression of an entire regulon, we wanted to record global proteomic data on ComK quantity and expression of the competence proteins in addition to the data on the *comG* reporter gene fusion. These time-resolved data reveal that there was a clear increase of ComK levels in all strains under control conditions during the transition into the stationary phase (Fig. 7a). The wild type, the Δ*sigB* mutant, and the *comK* complementation strain BAR609 even showed almost identical ComK intensities. Only in strain BAR610, significantly reduced intensities were observed for ComK, especially in direct comparison to strain BAR609. Notably, the only difference between these strains is the relocalization of the transcribed *comK* gene in BAR609, i.e. the separation of the transcription of *as-comK* and *comK*. Both BAR609 and BAR610 lack the negative control of SigB by RsbW, and thus even basal level expression of *sigB* (see Fig. 6a) leads to active SigB, which can trigger higher expression of SigB-dependent genes, such as *dps* and *ctc* (Fig. 6b) and also *yhxD-S365* (Fig. S8). Increased levels of *as-comK* in turn are responsible for the reduced levels of ComK observed in BAR610 even under control conditions without aTc (Fig. 7a). This pattern was observed not only for ComK amount but also for ComK activity, which was measured by determining the levels of the ComK-dependent proteins ComC and promoter activity of P_comGA_ (Fig. 7b; Fig. S6). As expected, the addition of ethanol stress to the wild-type strain suppressed induction and accumulation of ComK. In the Δ*sigB* control strain, compared with the wild type, a reduced but clear induction and accumulation of ComK was observed after the imposition of ethanol stress. The reduced increase in comparison to the control conditions might reflect the extra burden of ethanol stress in the Δ*sigB* mutant. Stress-free induction of SigB in strain BAR610 led to a significant reduction of ComK levels, whereas increases in ComK levels were only partially lost after aTc-mediated SigB induction in BAR609, probably because *as-comK* and ComK are produced in *trans*, and the negative effect of *as-comK* is less pronounced in the complementation strain.

The intensity patterns described for ComK are essentially matched by competence protein ComC, the synthesis of which strictly depends on the activity of ComK. In the wild type, ethanol stress-dependent suppression of ComK regulon expression was observed. This suppression was present to a lesser extent in the control strain Δ*sigB,* and the stress-free system also displayed a clear dependence on the expression of active sigma factor SigB for the downregulation of ComK and its activity. In particular, it should again be noted that the SigB effect in the *comK* complementation strain BAR609 was not as strong as for strain BAR610. The observations from the global mass spectrometry data are fully congruent with the results of the functional analyses of the transformation assays (Fig. 2 and 3) and confirm the notion that SigB has a direct negative effect on the expression and the cellular amount of ComK. The gradual differences between the strains BAR610 and BAR609 can be attributed to less efficiency in *trans* vs in *cis* action of the *as-comK* (S365).

## DISCUSSION

In the present study, we have revealed that the general stress response sigma factor SigB is able to reduce or prevent natural competence development upon activation by environmental stresses. On the molecular level, this block is accomplished by the SigB-dependent induction of the *comK* antisense RNA as-*comK* (S365). Interestingly, the chromosomal organization and transcriptional arrangement of the two operon structures consisting of *yhxD-as-comK* (S365)*-yhzC* and *comK*-S367-*yhjA* (Fig. 4a) correspond to the noncontiguous operon configuration discovered in *Staphylococcus aureus* by S. Sáenz-Lahoya et al. (31) and then substantiated in *S. aureus* and *E. coli* phages (35–37). In brief, this model states that two divergently transcribed overlapping operon mRNAs with internal complementary segments of non-coding regions to coding regions contribute to expression regulation by either modification of transcript stability (38–40) or transcriptional interference (41, 42). However, the transcriptional configuration of the chromosomal *yhjA-yhxD-comK-yhzC* region is particularly unique as it contains two overlapping non-contiguous operons. One is the *yhxD*-S365-*yhzC* operon, which is transcribed in a SigB-dependent manner and the other is the *comK*-S367-*yhjA* operon, which is autoregulated by ComK. It is noteworthy that these two non-contiguous operons overlap in the regions *yhxD*-S365 and *comK*-S367, with S365 representing the as-RNA for *comK* and S367 the as-RNA for *yhxD* (Fig. 4c). Therefore, the regulation of *comK* is due to the transcriptional organization of two non-contiguous operons, with S365 as the central region of the *yhxD*-S365-*yhzC* transcript representing an antisense transcript for *comK* (as-*comK*). Importantly, it was shown that transcription of the S365 RNA alone also leads to inhibition of *comK* expression (Fig. 5c). At least one interesting observation from the Northern blot studies (Fig. 4 signals 2a and 2b) already indicates the involvement of a yet unknown RNase, which cuts the RNA duplex produced in *cis* in the as-*comK* (S365) and in the 5'-region of the *comK* transcript. Nevertheless, this genetic configuration facilitates the mutual regulation of overlapping transcripts, offering an additional complicated but elegant mechanism for synchronizing gene expression.

From a physiological point of view, it has been shown that high level expression of the as-*comK* RNA is accompanied by a sharp decrease of *comK* mRNA levels resulting in reduced ComK protein levels and a failure of sufficient ComK target gene expression, resulting in a strongly impaired transformability. Notably, this effect is independent of the particular stress imposed and solely due to increased activity of SigB. Moreover, this stress-induced and SigB-mediated reduction of competence represents the first described daylight-inducible phenotype in *B. subtilis*. The conditions that favor competence of *B. subtilis*, namely high glucose levels and low amino acid supply, limit alternative responses, such as sporulation and the general stress response (Fig. 1). Here, we presented “darkness” as a novel competence-favoring determinant. Light as a SigB-inducing stimulus could represent a direct threat because of radiation-induced DNA damage or, rather, an indirect indicator of potential co-occurring stresses. In the natural habitat of *B. subtilis*, direct sunlight can also be considered as an indicator of present/future imminent heat, drought, and osmotic stress. Thus, early adaptation to this very likely stress would prime cells for improved survival by the synthesis of protective general stress proteins as well as simultaneous deactivation of unnecessary and costly differentiation programs.

The ability of SigB to prevent competence by limiting the amount of the critical competence transcription factor ComK represents an important link connecting two major adaptation processes in the regulatory network of *B. subtilis* that can in principle occur under the same environmental conditions. There are precedents for such interactions of the general stress response with other components of the regulatory network of *B. subtilis*. Sporulation can be impacted by SigB-mediated induction of the expression of the phosphatase Spo0E (17, 18), which then subsequently targets the phosphorylated form Spo0A-P of the master regulator of sporulation, Spo0A, and its dephosphorylation by Spo0E then blocks spore development under particular conditions (Fig. 1). Similarly, SigB can limit biofilm formation, an effect that is likely mediated by ensuring sufficient expression of the critical biofilm regulator SinR (43). Furthermore, biofilms of wild-type *B. subtilis* are more stress-resistant compared with those of isogenic *sigB* mutants, display more efficient dispersal, and swimming proficiency is more pronounced (43). Altogether, the common theme emerges that SigB has an important function in balancing resource allocation in such a way that, under severe life-threatening stress conditions, limited resources are channeled towards production of resistance-providing stress proteins by limiting the expression of alternative adaptive branches, such as sporulation, biofilm formation, and competence. Life-threatening stress situations might trigger very strong induction of the SigB response, which serves to provide high levels of stress proteins required to sustain life. In agreement with this idea, the SigB response is strongly activated after a rapid stress impulse but not, or only weakly, in response to a gradual increase of the stress signal (44). We propose that the resources for sufficient production of the general stress proteins in severely stressed cells can only be provided if SigB can interfere with complex signal transduction systems or gene regulation systems in order to block alternative survival programs, such as sporulation or competence in a state of emergency to save sufficient resources required for survival. This implies a hierarchy of adaptive responses in severely stressed cells. Further, we propose that competence development, biofilm formation, and sporulation are branches of last-resort adaptive mechanisms for starved cells, whereas the general stress response has a pivotal role for early stationary-phase adaptation and long-term survival in the state of a vegetative dormant and stress-resistant cell when physical stress is encountered. Finally, we believe that the identification and functional characterization of specific nodes connecting master regulators, as exemplified by SigB, Spo0A, and ComK of *B. subtilis*, in a given microbial signal transduction network will be of great importance to fundamentally understand the responses to environmental insults and to predict or deduce universal rules of microbial adaptation strategies.

## MATERIALS AND METHODS

### Generation of mutant strains

All modifications were introduced into the chromosome of *B. subtilis* 168. A modified two-step fusion PCR protocol (45) was used to generate a linear DNA fragment carrying the modified region together with a respective resistance marker gene, flanked by homologous wild-type sequences representing the chromosomal upstream and downstream regions of the mutation site. Newly created strains are described in detail below. If only chromosomal DNA of a described donor was used for the transformation of a described recipient, this is noted in the comments of Table S1. Schematic representations of the mutant strains BAR610, BAR606, and BAR609 can be found in Fig. S2 to S4. The BAR50 (Δ*yraA::phleo*) deletion mutant was constructed as in frame deletion, where the phleomycin resistance gene exactly replaces the coding region of *yraA*, but the wild-type transcription and translation regulation are preserved. The primers and templates used to create the three fusion fragments can be found in Table S2. The strain BAR70 carries a xylose-inducible copy of the T7 polymerase gene that was amplified with the primers (T7_pol_4_pX_for, T7_pol_4_pX_rev – Table S2) from template DNA of *E. coli* BL21 (DE3) and cloned into the integration vector pX1 (46) via the attached *Bam*HI sites. Linearized pX1-*T7 pol* was finally integrated into the chromosomal *amyE* locus of *B. subtilis* 168. To create strain BAR78, a spectinomycin resistance gene was cloned behind the gene *yhjA*. The *yhjA* gene is transcribed divergently to *yhxD*, whereby the monocistronic transcript is expressed from a constitutively active SigA promoter (U845) and terminated at the terminator D570 (27). The spectinomycin resistance gene was positioned together with its own translation initiation signal (Shine Dalgarno) as a transcriptional fusion behind the *yhjA* gene and in front of the terminator. This resulted in a bi-cistronic transcript (*yhjA-spec*) that leaves all factors of wild-type regulation untouched. The primers to generate the three fusion PCR fragments are shown in Table S2. The fusion construct was used to transform the recipient strain BAR71. For the construction of strain BAR85, chromosomal DNA from strain BAR78 was used as a starting point. With the four primers (comK_up_for; T7_in_rev) and (T7_in_for; yhjB_do_rev), two fusion fragments were created by PCR, which were used to exchange the SigB promoter of *yhxD* by a T7 promoter. The T7 promoter also contained a XylR binding site to minimize unwanted basal expression. Thus, a derepression of the cloned target T7 promoter was performed exclusively together with the derepression of the *xylA* promoter for transcription of the T7 polymerase gene in the *amyE* locus by the addition of xylose. This arrangement allowed the T7-dependent transcription of the *yhxD* mRNA and the *comK* antisense transcript *as-comK* (S365). The fusion construct was used to transform the recipient strain BAR71. To create strain BAR79, chromosomal DNA from strain BAR78 was also used, and two fusion fragments were created with the four primers (comK_up_for; T7_in_comK_fusion_rev) and (T7_in_for; yhjB_do_rev). These two fragments also contained a substitution of the *yhxD* SigB promoter with the T7 promoter and a deletion of the entire *yhxD* reading frame together with the *yhxD* terminator (D568-D569)(27). The fusion construct was used to transform the recipient strain BAR71. This arrangement allowed the T7-dependent transcription of the *comK* antisense transcript *as-comK* alone. All mutants were selected on LB agar plates containing the respective antibiotics at the final concentration as follows: erythromycine/lincomycine (5 µg/mL / 25 µg/mL), spectinomycin (200 µg/mL), tetracycline (17 µg/mL), chloramphenicol (5 µg/mL), phleomycin (5 µg/mL), or kanamycin (5 µg/mL). Chromosomal DNA of each mutant was sequenced to verify the correct mutation.

### Culture conditions

Unless otherwise stated, all experiments have been carried out at least as biological triplicates. All bacterial culturing was performed in a darkened laboratory under exclusion of daylight with a weak artificial light source. Light sources in the shaker were deactivated, and viewing windows were sealed with aluminum foil. Glass flasks for the cultivation of the main cultures were also wrapped with aluminum foil up to the top edge. For inoculation of main cultures in competence medium (47), pre-cultures in lysogeny broth (LB) were grown overnight. Therefore, in the evening before the experiment, 12 disposable reagent tubes with 5 mL LB each were prepared for each strain, and 300 µL was transferred from a glycerol stock culture into the first tube. From the first tube, a dilution of 1.4:10 was prepared by sequentially transferring and mixing 700 µL into the next tube. This bacterial dilution series was cultured in an air incubator at 37°C and 220 rpm overnight under light exclusion (maximum 10 h). In the next morning, the optical density of the last dilutions of the overnight culture was measured at 540 nm and an exponentially growing culture (OD_540_ > 0.6 and < 1.0) was selected to inoculate the main culture in competence medium to a start OD of 0.05 at 500 nm. Therefore, the required volume of the overnight culture was transferred into a preheated 15 mL (37°C) falcon tube, and the cells were pelleted quickly and gently for 45 s at 2,236 × *g* (~5000 rpm) in a centrifuge with pre-heated rotor (37°C). The supernatant was completely removed and discarded, and the cell pellet was suspended in the preheated (37°C) competence medium of the main culture and incubated at 220 rpm under exclusion of light in an air incubator at 37°C. Routinely, 500 mL flasks with 60 to 100 mL of medium were used for all experiments. The growth was documented by measuring the optical density at 500 nm, and samples for the quantitative transformation assays, the FACS, and Western and Northern blot experiments as well as mass spectrometry were taken at the specified ODs.

### Cell sampling and anhydrotetracycline or xylose induction

As soon as the cell cultures reached an optical density of OD_500_ = 1.6, 8 OD units for RNA and protein samples were taken, cooled down with liquid nitrogen, and centrifuged at 4°C and 8,500 rpm for 3 min. Subsequently, 0.3% xylose (wt/vol) or 20 ng/mL anhydrotetracycline (aTc) for the respective strains were added, and the flasks were further incubated at 37°C and 220 rpm. Additional samples were taken after 20 and 40 min according to the previous one.

### Isolation of total RNA

Harvested cells were resuspended in 200 µL lysis solution (4 M guanidine-thiocyanate, 0.025 M Na-acetate [pH 5.2], 10% [wt/vol] N-lauroylsarcosinate) and immediately transferred to a precooled (liquid nitrogen) 4.8 mL Teflon vessel containing an 8-mm diameter steel ball. Cells were mechanically disrupted using a Dismembrator (Retsch GmbH, Haan, Germany) at 2,600 rpm for 3 min. Cell powder was resuspended with 2 mL lysis solution and immediately cooled in liquid nitrogen. Subsequently, total RNA was isolated according to the acid phenol method of Majumdar et al. (48). RNA samples were frozen and thawed three times (2 min on liquid nitrogen and 2 min at 40°C) to achieve properly dissolved RNA.

### Quantitative near-infrared (NIR) Northern blot analysis

NIR Northern blots were performed according to the protocol of ProTec Diagnostics GmbH. To determine transcript sizes, the RNA TRUE Ladder from ProTec Diagnostics GmbH was used. RNA blots were methylene blue stained to check for RNA quality and equally loaded amounts. RNA probes for *yhxD*, *yhzC,* and *comK* were biotin labeled by *in vitro* transcription with T7 RNA polymerase from gene-specific PCR products fused to a T7 promoter. The primers used for generation of the respective T7 templates are listed in Table S2.

### Quantitative transformation assays

For the transformation assays, the respective strains were grown under control conditions in the dark (darkness control), until cultures reached an optical density of OD_500_ = 1.6, where the cells were either exposed to a True Light LED source for 60 min or stressed by the addition of 2% (vol/vol) ethanol, or induced by the addition of 20 ng/mL anhydrotetracycline (aTc). When cultures reached an OD_500_ of 2.0, 4 mL of the cultures was transferred to a fresh prewarmed 100 mL glass flask and mixed with 400 ng of chromosomal DNA of a Δ*yraA* mutant (BAR50) carrying a phleomycin resistance gene (Fig. 2), 400 ng of chromosomal DNA of BAR619 (*ctsR-mcsA-mcsB-clpC-Km*) carrying a kanamycin resistance gene (Fig. 3a and c), or 1,250 ng of PDG148 plasmid DNA (Fig. 3b) pDG148 (49). For control experiments, 4 mL of the cultures was transferred to a flask without added DNA. All cultures were then incubated for 1 h at 90 rpm and 37°C. Then, 2 mL of fresh pre-warmed LB medium was added, and cultures were incubated for another 2 h at 220 rpm and 37°C. Subsequently, serial 1:10 dilutions of the cultures were prepared up to a 10^−8^ dilution in LB at room temperature. Then, 100 µL aliquots of the dilutions were plated in two technical replicates on LB plates to determine the colony forming units (CFU) as well as selective LB plates containing either 5 µg/mL phleomycin or kanamycin to determine the numbers of transformed colony forming units (TCFU) using plastic Drigalski spatulas. All transformation assays were performed for a minimum of three biological replicates per strain. Transformation frequencies (η*S*) were determined by normalization to the viable counts (CFU) of each strain and experiment using the formula η*S* = *Cr*/(*C_T_ pD*), where *C_r_* is the average number of TCFU, *C_T_* is the average total number of CFU, and *pD* is the DNA concentration in micrograms per milliliter, from biological replicates (50). Transformation frequencies were then normalized (set to 100%) to the respective positive control strain showing the highest transformation efficiency for the respective experimental setup and enabling direct comparison of the strains or conditions tested. We would also like to emphasize that we have improved the generation of competent subpopulations and transformation in *B. subtilis* in general. Previous reports on competence population dynamics vary from 10 to maximally 20% of cells entering the competence state (51–53). Here, we could show that cultivation protected from light exposure already caused an increase of the competent population to ~50% in the *B. subtilis* wild-type strain and improved transformability rates by more than one order of magnitude. Moreover, elimination of the negative effector SigB further enhanced this effect, resulting in a competent population of ~65% and a further 2.5-fold increase of transformation frequencies observed for the *sigB* mutant. Additionally, a synchronized timing of ComK activation as well as competence gene expression could be achieved by the use of pre-cultures in LB and subsequent inoculation of the competence promoting medium with exponentially growing cells as described in detail in the Materials and Methods. Using this procedure, competence development was precisely induced when cultures reached an OD_500_ of 1.6 and reached maximal levels at ~60 min after entry into the stationary phase.

### Light exposure

For the light stress experiments, a full-spectrum daylight LED of the manufacturer True Light International GmbH (article number 8012) was used. According to the manufacturer’s specifications, the LEDs have a nominal luminous flux of 970 lumens at 25°C, a correlated color temperature (CCT) of 5500 Kelvin and a color rendering index (CRI) of 96 (%). The light source (LED carrier plate) has been spatially separated from the voltage transformer in the bulb to avoid possible heat influence on the cells. The shaking flasks used had a base diameter of 13 cm and a height of 23 cm. The neck of the flasks had a diameter of 7 cm. The LED carrier plate was connected to the lid of the shaking flask and was positioned inside the flask with a distance of 15 cm from the bottom. With the nominal light beam of 970 lm, a solid angle of about 45°, and a distance of 15 cm from the culture surface, an illuminance (E) of about 90,138 lx was achieved on the bottom surface of the shaking flask (area of 133 cm^2^). This value corresponds to the average illuminance of ~90,000 lx on the surface of the earth by the sun under a clear sky and a sun elevation angle of 60° (Central Europe at noon in summer) (54).

*I* = lm/sr (luminous intensity [*I*, candela {cd}] = luminous flux [lumen {lm} divided by solid angle [steradian {sr}]); *E* = *I*/*d*² (illuminance [*E*, lux {lx} = luminous intensity [*I*, candela {cd}] divided by radius² or distance²).

### Growth conditions and cell sampling of the GFP-positive *B. subtilis*

The strains BAR78, BAR85, and BAR79 were grown under control conditions in the dark (darkness control) or in the dark until cultures reached an optical density of OD_500_ = 1.6. Then, the cells were either grown without xylose or with addition of 0.3% xylose (wt/vol) to induce the expression of the T7 polymerase and to de-repress the T7 target promoter where applicable. GFP expression was monitored throughout growth by fluorescence-activated cell sorting (FACS), and samples were taken at specific optical densities (OD, 0.8, 1.2, 1.6, and 2.0) and 60, 120, 180, and 240 min after entry into the stationary phase (OD 2.0).

### Fluorescence-activated cell sorting (FACS) analysis

Bacterial cells (300 µL culture volume) were sampled on ice and analyzed immediately in a FACSAria III high-speed cell sorter (Becton Dickinson Biosciences, San Jose, CA, USA) with 488 nm excitation from a blue Coherent Sapphire solid-state laser at 18 mW. Optical filters were set up to detect the emitted GFP fluorescence signal at 530/30 nm (FITC channel). Samples were prepared by using 100 µL bacterial culture suspension diluted with 1 mL PBS. All data were recorded at logarithmic scale with the FACSDiva v6.1.3 software (Becton Dickinson). Prior to measurement of experimental samples, the proper function of the instrument was determined by using the cytometer setup and tracking software module (CS&T) together with the CS&T beads (Becton Dickinson Biosciences). First, in a SSC-area versus FSC-area dot plot, the bacterial cell population was gated. The detection thresholds and photomultiplier (PMT) voltages were adjusted by using a non-GFP-expressing strain (BAR 502). The GFP signal from the scatter gate population was monitored in a GFP-area histogram. For each sample, 10.000 events in the scatter gate were recorded.

### Quantitative near-infrared (NIR) Western blot analysis

Five micrograms of the protein lysate was allocated to each lane and separated by using SDS-PAGE with Mini-Protean. Subsequently, the proteins were transferred to a polyvinylidene difluoride (PVDF) membrane for Western blot analysis as described previously (55). The NIR Western blot detection was performed according to the protocol of LI-COR (Lincoln, NE, USA)(56). The polyclonal antibody against SigB (57) was diluted 1:5,000.

### Proteome analysis

To obtain protein lysate, cells were mechanically disrupted as described previously (58). Cell powder was solubilized in 20 mM HEPES (pH 8.0) with 1% (wt/vol) SDS. The lysate was transferred into a fresh pre-lubricated 1.5 mL low binding Eppendorf tube and incubated for 1 min at 95°C and 1,400 rpm. After cooling down to RT, 2 µL of a 1 M MgCl_2_ stock solution (final conc., 4 mM MgCl_2_) was added. Subsequently, 1 µL of a 1:100 diluted benzonase (Pierce Universal Nuclease; Pierce) stock solution (final 0.005 U/µL) was added and incubated for 15 min at 37° and 1,400 rpm. The samples were placed in an ultrasonic bath at RT for 5 min until the thick lysate became a fluid due to the complete breakdown of DNA and RNA. The protein lysates were centrifuged for 30 min at 17,000 × *g* and transferred into a fresh 1.5 mL low binding Eppendorf tube, while the cell debris pellet was discarded. Automated protein concentration determination and sample digestion for mass spectrometry analysis were performed following the protocol of Reder et al*. (58*) by using the modified OT-2 liquid handling robot (Opentrons, Long Island City, NY). For the LC-MS measurement, the UltiMate 3000 RSLC nano System (Thermo Fisher Scientific) was coupled to an Orbitrap Exploris 480 mass spectrometer (Thermo Electron, Bremen, Germany). Separation of tryptic peptides was achieved by an Accucore 150-C18 analytical column (25 cm × 75 µm, 2.6 µm C18 particles, 150 Å pore size, Thermo Fisher Scientific) at a constant temperature of 40°C and flow rate of 300 nL/min. Analysis with the Exploris was conducted in data-independent mode with following specifications: 350–1,200 *m*/*z* scan range, 120,000 MS and 30,000 MS/MS scan resolution, AGC target of 3e6 (300% MS, 100% MS/MS), 66 isolations windows, 13 *m*/*z* isolation window width, 30% HCD collision energy, and 2 *m*/*z* overlap windows. Detailed information on the LC gradient and additional parameters for MS/MS analysis are shown in Table S6.

### Analysis of mass spectrometry data

Spectronaut version 17 (Biognosys AG) was used in order to analyze the data-independent (DIA) proteome experiment using the database Uniprot/Swissprot for *B. subtilis* with 4,198 entries including isoforms (version 2021_01). Settings of the Spectronaut search can be found in Table S4. The whole DIA-MS analysis was performed using R (R version 4.2.0 [22 April 2022]) (59) and tidyverse package (version 1.3.2) (60) with R functions conceived and written by S. Michalik. The raw data output from Spectronaut was used to perform a median normalization (if normalization in Spectronaut was unchecked) over the MS2 total peak area intensities (EG.TotalQuantity) or use the Spectronaut normalization method if selected. Zero intensity values were replaced using the half-minimal intensity value from the whole data set. Methionine oxidized peptides were removed from the analysis. To generate peptide intensity data, the sum over ions per sample and peptide was calculated. iBAQ protein intensities were extracted from the Spectronaut report.

### Statistics

Statistical significance of differences was determined using a two-sample *t*-test with at least three biological replicates. *P*-values were adjusted according to Benjamini Hochberg and coworkers (61) and an adjusted *P *< 0.05 was considered significant: n.s., *P* > 0.05; *, *P* ≤ 0.05; **, *P* ≤ 0.01; ***, *P* ≤ 0.001. Statistics can be found in supplemental information.

## SUPPLEMENTAL MATERIAL

Supplemental Figures and Tables mbio.02274-24-s0001.pdf Fig. S1–S8 and Tables S1–S6.

Supplemental Statistics mbio.02274-24-s0002.xlsx Statistical tests.

ASM does not own the copyrights to Supplemental Material that may be linked to, or accessed through, an article. The authors have granted ASM a non-exclusive, world-wide license to publish the Supplemental Material files. Please contact the corresponding author directly for reuse.
