# Supplementary material for: Activation of the general stress response sigma factor SigB prevents competence development in Bacillus subtilis
Source: mBio. 2024 Oct 29;15(12):e02274-24. doi: 10.1128/mbio.02274-24 (PMC11633097; doi:10.1128/mbio.02274-24)
Supplement: Supplemental Figures and Tables — Fig. S1–S8 and Tables S1–S6. [file mbio.02274-24-s0001.pdf]

Supplementary Information

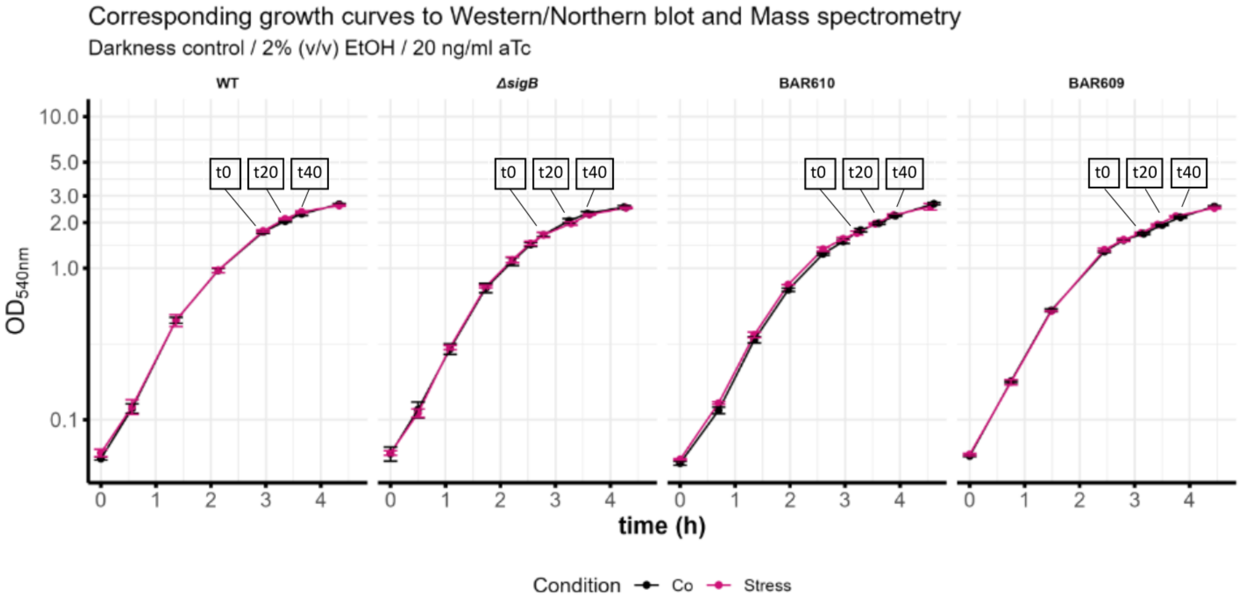

Supplementary Figure S1. Growth curves of cultures used for Western/Northern blot analysis and proteome profiling by mass spectrometry.

The main cultures of all strains were grown in competence medium as described in materials and methods. In brief, a required volume of an exponentially grown LB pre-culture was transferred into a tempered 15 ml (37°C) falcon tube in order to inoculate the main culture to a starting OD_{500} of 0.05. The cells were immediately and gently pelleted by centrifuging for 45 s at 2236 g (~ 5000 rpm) in a pre-warmed rotor (37°C). The supernatant was completely removed and discarded and the cell pellet was suspended in the preheated (37°C) competence medium of the main culture and incubated at 220 rpm under exclusion of light in an air incubator at 37°C. Two samples were taken from control cultures grown in competence medium without ethanol or aTc in the late exponential growth phase (OD_{500} = 1.6 t0) and 20 min later (t20) as well as 40 min (t40) later (only for Western blot and mass spectrometry) in transient phase. For the ethanol stress and stress-free aTc induction an identical control sample was harvested at OD_{500nm} = 1.6 (t0) right before addition of 2% [v/v] ethanol for the wild-type and the isogenic Δ*sigB* mutant or 20 ng/ml aTc for the strains BAR610 and BAR609 and 20 min (t20) as well as 40 min (t40, only for Western blot and mass spectrometry) after SigB induction.

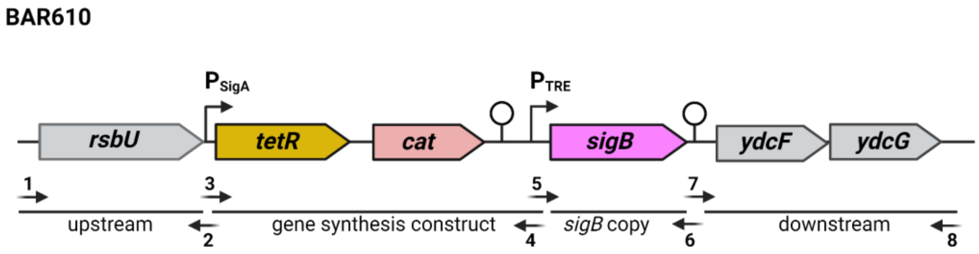

Supplementary Figure S2. Generation of strain BAR610.

A generally applicable regulatory construct was created using gene synthesis (GenScript Treubstraat 1, 1st floor. 2288EG, Rijswijk, Netherlands). The synthesized gene product consisted of the gene for the tetracycline repressor under the control of a constitutively active SigA type promoter and chloramphenicol resistance cassette. The transcriptional fusion of these two genes is followed by a strong terminator to prevent read-through into downstream genes or the PTRE promoter region. Downstream of the terminator, a SigA type promoter was localized under TetR control. The primers used to assemble the transformation construct are numbered consecutively and listed in Table 2.

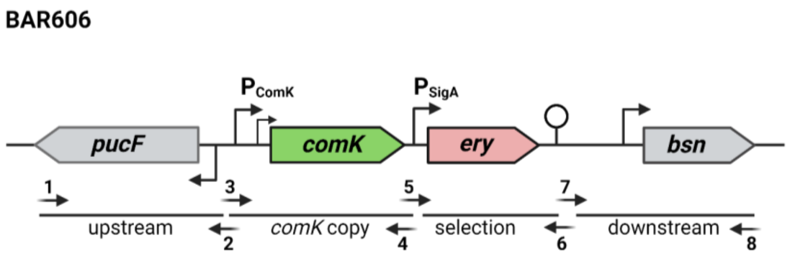

Supplementary Figure S3. Generation of strain BAR606.

The *comK* gene was cloned together with its native regulatory upstream sequence (two SigA type promoters), an erythromycin resistance cassette as well as a strong terminator between the divergently transcribed genes *pucF* and *bsn*. The selection of this integration site was based on its transcriptionally inactive nature in this region (1), ensuring the greatest possible distance to the original *comK* locus. A transcriptional fusion occurs from the two *comK* promoters and the dicistronic operon *comK-ery* is transcribed (approximately 1600 b). For selection under control conditions, the erythromycin cassette was expressed solely from a constitutively active promoter. The primers used to assemble the transformation construct are numbered consecutively and listed in Table 2.

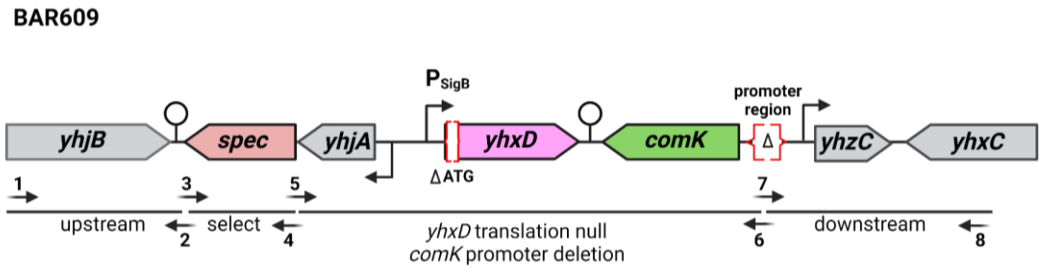

Supplementary Figure S4. Generation of strain BAR609.

A deletion of the *comK* promoter would generate a strain that is no longer transformable due to the lack of positive autoregulation of ComK. Therefore, to generate the strain BAR609, strain BAR606 was first transformed with the construct from strain BAR610 to generate a strain with SigB under the control of P_{TRE} and a copy of *comK* in *trans*. Then, the *comK* promoter deletion fragment, as shown here, was transformed. For selection, a spectinomycin resistance cassette was inserted as a transcription fusion downstream of the constitutively expressed *yhjA* gene. In addition, the START codon (ATG) of *yhxD* (translation null mutant) and the native promoter region of *comK* were deleted (transcription null mutant). These two mutations were introduced to ensure that the SigB-dependently expressed protein YhxD does not have an effect on competence formation and to check whether a *comK* anti-sense RNA can have an effect in *cis* or possibly also in *trans*. The primers used to assemble the transformation construct are numbered consecutively and listed in Table 2.

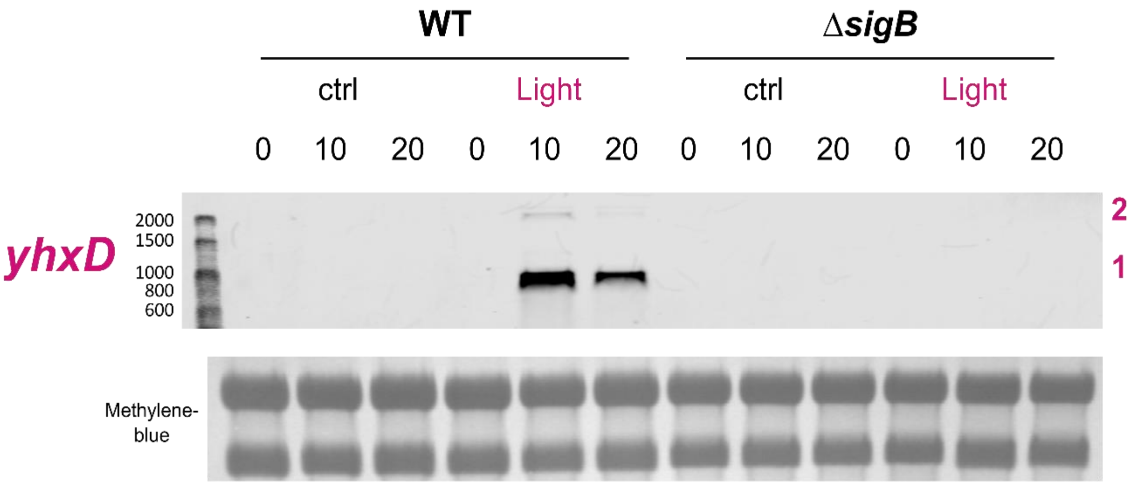

Supplementary Figure S5. Light induction of the *yhxD* gene of *B. subtilis*.

Northern blot experiments displaying the expression profile of the *yhxD* transcript under control conditions (ctrl; darkness controls) and 10 min (t10) as well as 20 min after turning on the light for the wild-type strain and the *sigB* mutant. Cells were grown in competence medium in the dark and the light source integrated into the special flask was switched on when cultures reached an optical density of OD_{500nm} = 0,4 (early logarithmic growth phase). Detected transcripts are numbered and color-coded according to the schematic presentation in Fig. 4a. Methylene blue staining served as a RNA loading control.

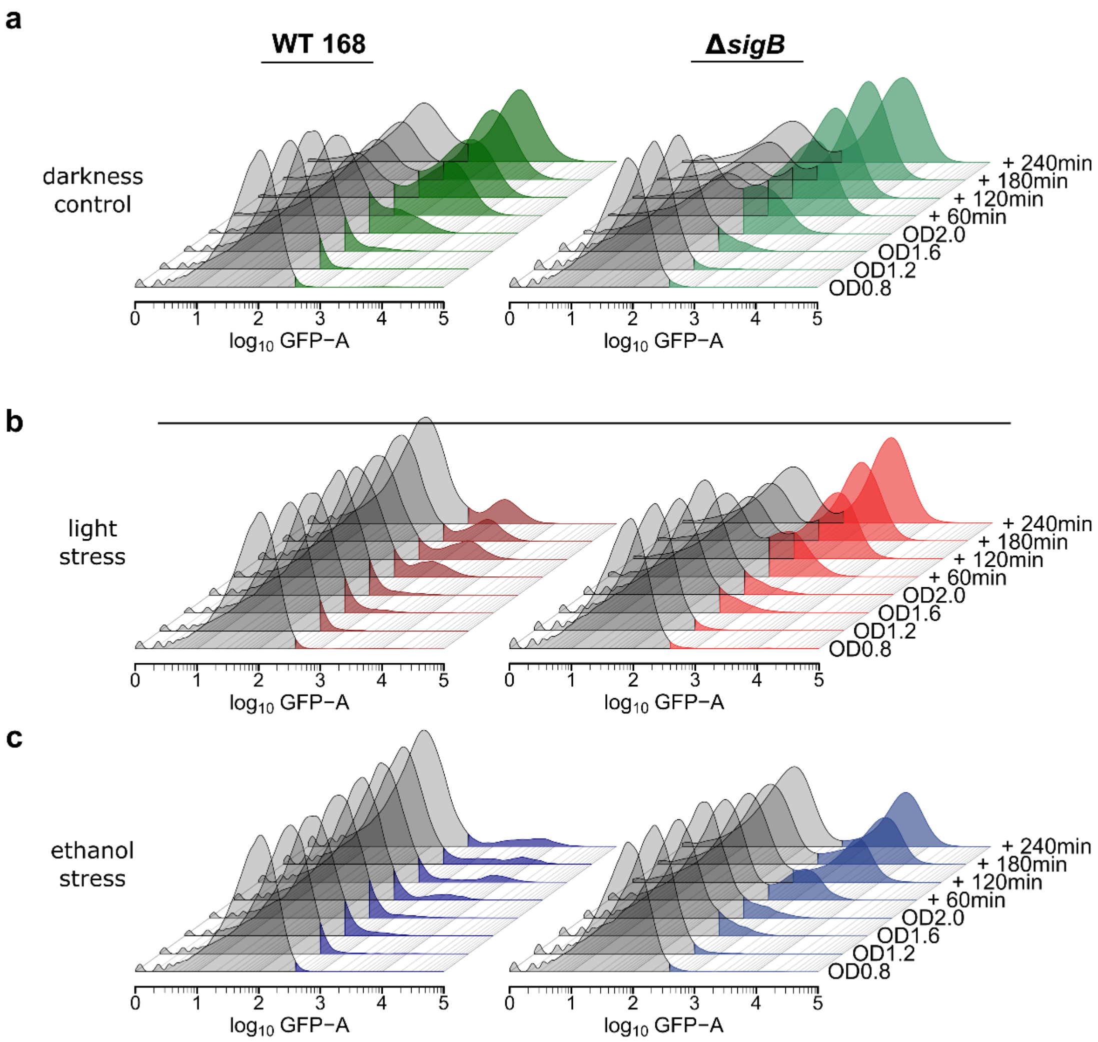

Supplementary Figure S6. Monitoring of ComK-dependent competence promoter activity.

The expression of the *comG* operon is strictly dependent on activation by ComK and therefore serves as an indicator for cells entering the competence state (2). Therefore, FACS population dynamic measurements by single cell analysis of a P_{comG}-GFP reporter gene fusion were performed for the *B. subtilis* WT 168 and its isogenic Δ*sigB* mutant under control conditions (darkness, **a**), after exposure to a True Light LED source for 60 min (**b**) as well as after exposure to 2% ethanol stress (**c**). Samples were taken at specific optical densities (OD 0.8, 1.2, 1.6, 2.0) and 60, 120, 180 and 240 min after entry into stationary phase (OD 2.0). The non-competent populations are shown in grey and the competent populations are colored in green (control), red (light stress) and blue (ethanol stress).

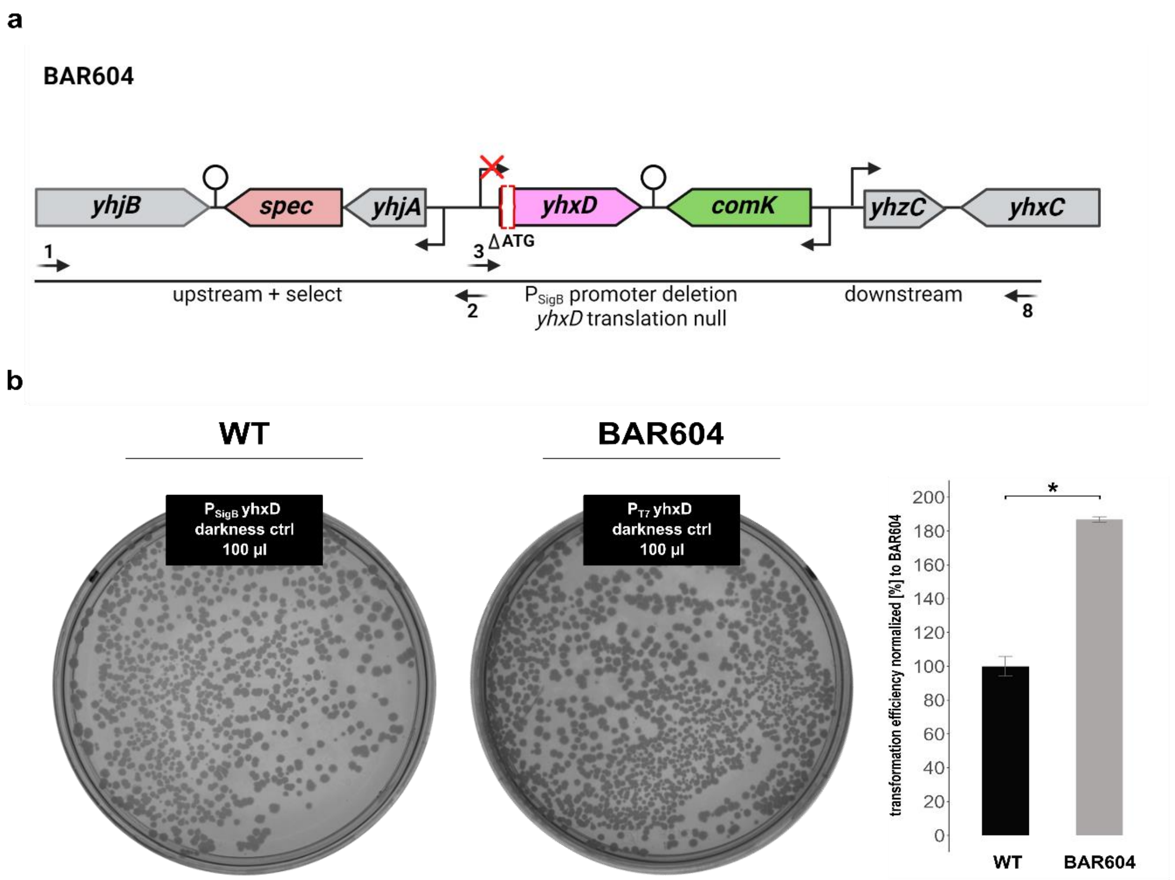

Supplementary Figure S7. Generation of hypercompetent strain BAR604 and quantitative transformation assay.

a) Schematic representation of the hypercompetent strain BAR604. The SigB promoter of *yhxD* was deleted, resulting in a silencing of the *yhxD-as-comK*(S365). For selection, a spectinomycin resistance cassette was inserted as a transcription fusion downstream of the constitutively expressed *yhjA* gene, which was already generated in BAR609. The primers used to assemble the transformation construct are numbered consecutively and listed in Table 2. **b)** To determine transformation efficiencies, dilution series were plated onto both LB agar plates and selective LB agar plates to count the total colony forming units (CFU) and the transformed colony forming units (TCFU) after overnight incubation (24 h) at 37°C. Transformation frequencies (in percent) were determined by normalization on the basis of the viable counts (CFU). Two representative selective agar plates of replicate genetic transformation experiments of the *B. subtilis* wild-type 168 and BAR604 are shown. Quantitative data from at least three independently performed biological replicates of the transformation assays are given as bar plots with the respective standard

deviations. The transformation frequencies of BAR604 were normalized to the *B. subtilis* 168 strain. Statistical significance within the bar plots is represented by p-values (*) <= 0.05.

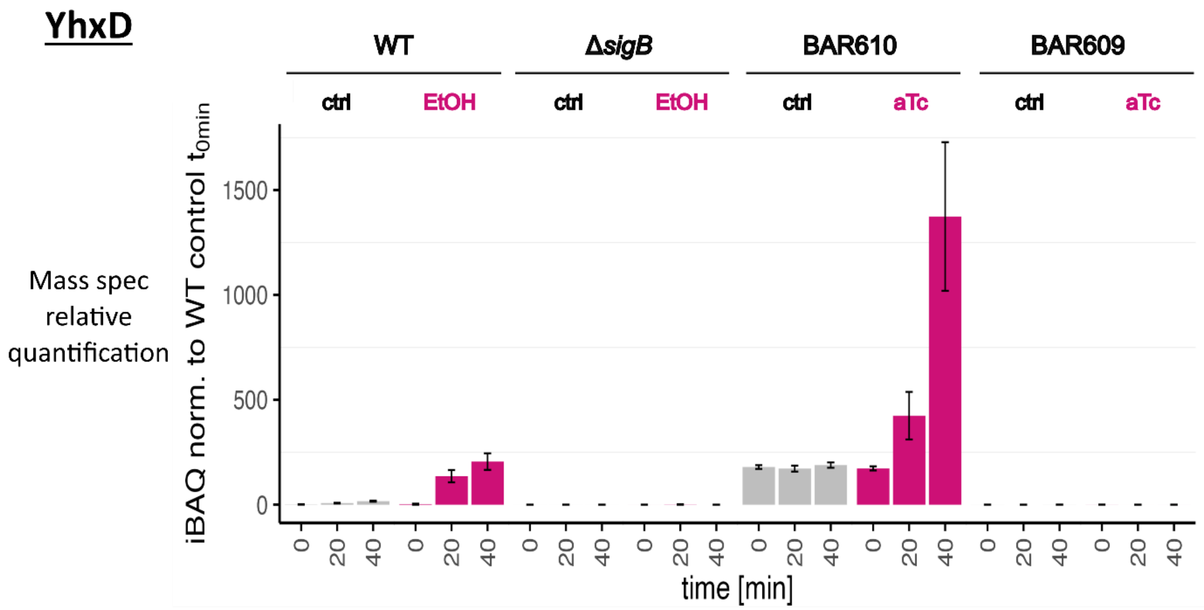

Supplementary Figure S8. Time resolved proteome profiling of YhxD in the respective *B. subtilis* strains. Bar plots of the iBAQ intensities of the SigB dependent YhxD protein, with standard deviations from a DIA-MS analysis are shown. Three independent biological replicates were measured. The respective quantities were normalized against the wild-type sample at control time t0.

Supplementary Table S1. Bacterial strains

Strain	Relevant features	Reference	Comment
B. subtilis 168	*trpC2*	(3)	
B. subtilis BSB1	*trp*$^{+}$	(1)	
B. subtilis ML6	*trpC2*, *sigB::*ΔHindIII–EcoRV::*cat*	(4)	
B. subtilis P*comG*-gfp	*trpC2*, P*comG*::*gfp* (km)	(5)	
B. subtilis BAR50	*trpC2*, Δ*yraA::phleo*	This study	primers in table 2
B. subtilis BAR78	*trpC2*, *PcomG::gfp* (km), *amyE::*P*xyl-T7-pol* (cm), *yhjA::spec*	This study	primers in table 2
B. subtilis BAR79	*trpC2*, P*comG::gfp* (km), *amyE::*P*xyl-T7-pol* (cm), *yhjA::spec* ΔP*sigB*::P*T7* Δ*yhxD*-Dterminator	This study	primers in table 2
B. subtilis BAR85	*trpC2*, P*comG::gfp* (km), *amyE::*P*xyl-T7-pol* (cm), *yhjA::spec* ΔP*sigB*::P*T7*	This study	primers in table 2
B. subtilis BAR604	*trpC2, Spec-yhjA-PT7 ΔATG yhxD*	This study	primers in table 2
B. subtilis BAR606	*trpC2, pucF-comK-ery-bsn, ΔrsbV-rsbW-sigB-rsbX::tetR-cm- PTRE-sigB*	This study	primers in table 2
B. subtilis BAR609	*trpC2, pucF-comK-ery-bsn, ΔrsbV-rsbW-sigB-rsbX::tetR-cm- PTRE-sigB, Spec-yhjA-ΔATG yhxD-ΔPcomK*	This study	primers in table 2
B. subtilis BAR610	*trpC2, ΔrsbV-rsbW-sigB-rsbX::tetR-cm- PTRE-sigB*	This study	primers in table 2
B. subtilis BAR619	*trpC2, mcsA-mcsB-clpC-Km*	This study	primers in table 2
Escherichia coli BL21(DE3)	str. B F$^{–}$ ompT gal *dcm lon hsdSB*(*rB*$^{–}$*mB*$^{–}$) λ(DE3 [*lacI* l*acUV5*$^{-}$T7*p07 ind*^{1} *sam*^{7} *nin*^{5}]) [*malB*$^{+}$]K^{-12}(λ^{S})	(6)	amplification of the T7 polymerase gene

Supplementary Table S2. Primers

Purpose	Name	Sequence (5´−3´)
BAR50 (Δ*yraA*)	yraA_up_for	CGATGGGTGCAGAAGTAACC
	yraA_up_rev	GCCGGGATAGACTGTAACATAAAAAACCCTCCTAATAACTC
	Phleo_just_for	ATGTTACAGTCTATCCCGGC
	Phleo_just_rev	TTAGCTCTTGATCTGTTGGAAG
	yraA_do_for	CTTCCAACAGATCAAGAGCTAACAAGAAGGCACAGACTGTTC
	yraA_do_rev	GGTATGGGGAACGACACCTTT
BAR70	T7_pol_4_pX_for	GGATCCATGAACACGATTAACATCGC
	T7_pol_4_pX_rev	GGATCCTTACGCGAACGCGAAGTCCG
BAR78	comK_up_for	CAATCTATCGACATATCCTGC
	yhjA_spec_fusion_rev	CGTGTTCATTCATGGACCTCCTTTTACCGTTCACAGGCGATAAG
	Spec + SD for	AAGGAGGTCCGTGAATGAACACGTACGAGCAGATC
	Spec_Just_rev	TTACAACTTCTTTAAGCGGTTGTTC
	yhjB_spec_fusion_for	GAACAACCGCTTAAAGAAGTTGTAAAGTAAGAAAAAGCAATCTGGTGTCCAGATTGCTTTTTTTATGCTTAGGCCGCCTGATCGGCCGCAGCG
	yhjB_do_rev	CAAAGTGTGGCCTGGTTCAG
BAR85	T7_in_for	CCAATAAACTAATCTCCCTATAGTGAGTCGTATTATGTTTTGGCAGTTTGAATAGTG
	T7_in_rev	CTATAGGGAGATTAGTTTATTGGATAAACAAACTAACACCAAACAGAAAAGGAGCG
BAR79	T7_in_comK_fusion_rev	CCAATAAACTAATCTCCCTATAGTGAGTCGTATTATGTTTTGGCAGTTTGAATAGTG
BAR610	sigB_TetR_PsigA-TRE_up_for_(1)	AAGCCATCAAATGGCTTCGG
	sigB_TetR_PsigA-TRE_up_rev_(2)	GTTTACGTTTGCGTGCCAATTCCTAGACTTTTCTCCGCAAAACAATTAACG
	reamp_sigB_TetR_PsigA-TRE_for_(3)	GAATTGGCACGCAAACGTAAAC
	TetR_TRE_just_rev_(4)	CTATCTTAATTATATCTCTATC
	sigB_BAR610_for_(5)	GATAGAGATATAATTAAGATAGTAAAGGAGGATAATCTAATGACACAACCAATCAAAAACTACGAAACTAAC
	reamp_sigB_TetR_PsigA-TRE_rev_(6)	TTACATTAACTCCATCGAGGG

	sigB_TetR_PsigA-TRE_do_for_(7)	CCCTCGATGGAGTTAATGTAACCTGACCTATATTCTCGGAC
	sigB_TetR_PsigA-TRE_do_rev_(8)	TTTTCCTACTTTCCGCTGCC
BAR606	comK_reloc_up_for_(1)	ATGTCAGACGGAGCTCATCC
	comK_reloc_up_rev_(2)	AACATCGCCTCTCCAGCTTAG
	comK_reloc_for_(3)	CTAAGCTGGAGAGGCGATGTTAATGCCTTTTTATAGTATATGGATAACGG
	comK_reloc_Cm_rev_(4)	CTGCCAAAGCATAATGGGATCTAATACCGTTCCCCGAGC
	Ery_+SigAPr_for_(5)	ATCCCATTATGCTTTGGCAG
	yfhD_Terminator_rev_(6)	TTATCCTGACGAGGCGCC
	comK_reloc_do_for_(7)	GGCGCCTCGTCAGGATAACGCGGAACGGATGAAAACAG
	comK_reloc_do_rev_(8)	GGTCGATAAATGGATTGCGG
BAR609	yhjA_up_for_(1)	GGACATCATGATTCTGGTTG
	yhjA_Spec_in_up_rev_(2)	GAACAACCGCTTAAAGAAGTTGTAAATAAGAAAAAGCAATCTGGTGTCCAGATTGCTTTTTTATGCTTAGGCCGCCTGATCGGCCGC
	Spec_+yhjA_Term_rev_(3)	GCGGCCGATCAGGCGGCCTAAGCATAAAAAAAGCAATCTGGACACCAGATTGCTTTTTCTTATTTACAACTTCTTTAAGCGGTTGTTC
	yhxD_-ATG_Start_rev_(4)	CGCTCCTTTTCTGTTTGGTG
	yhxD_-ATG_Start_for_(5)	CACCAAACAGAAAAGGAGCGTCTGATTCAAATCTTACGAATCC
	comK_promoter_removal_rev_(6)	CCTGTCGTAGAAAAAGGATGGAGGCCATAATATG
	comK_promoter_removal_for_(7)	CCATCCTTTTTCTACGACAGGTTTTTTAACATTTAAG
	comK_pro_deletion_rev_(8)	CCAGCTGATCAGAACCTTCC
BAR604	Primer (1)	Identical to (1) in BAR609
	yhxD_T7_Pr_in_rev_(2)	GGCAGTTTGAATAGTGAAGG
	yhxD_T7_Pr_in_for_(3)	CCTTCACTATTCAAACTGCCAAAACATTCCCTATCAGTGATAGAGAAATTAATACGACTCACTATAGGGAGATGTACACCAAACAGAAAAGGAG
	Primer (8)	Identical to (8) in BAR609
anti-*yhxD* Probe	yhxD_NOR_for	GAGGAACAGCCAGATGCTG
	yhxD_T7_rev	GAAATTAATACGACTCACTATAGGGAGAATGCCTTTGGAAGCAAGCTG
anti-*comK* Pobe	comK_NOR_for	AGTGAACGGCGCAACAATTG
	comK_T7_rev	GAAATTAATACGACTCACTATAGGGAGAGGAATTTGGTTCTGAGCCAC
	yhzC_NOR_for	CGTACACTGAGCTCATGAAG

anti-*yhzC* Probe	yhzC_T7_rev	GAAATTAATACGACTCACTATAGGGAGAGCTTATTCGCTCTGCTTGAG

T7 promoter sequences for *in vitro* transcription are underlined.

Supplementary Table S4. Transformation efficiencies.

Strain	Condition	Transf. frequency	Standard deviation
B. subtilis 168	control	$1,43 \times 10^{-4}$	$1,69 \times 10^{-5}$
B. subtilis 168	ethanol	$1,22 \times 10^{-5}$	$1,46 \times 10^{-6}$
B. subtilis 168	light	$3,29 \times 10^{-6}$	$2,38 \times 10^{-7}$
Δ*sigB* mutant	control	$3,82 \times 10^{-4}$	$2,81 \times 10^{-5}$
Δ*sigB* mutant	ethanol	$9,43 \times 10^{-5}$	$1,13 \times 10^{-5}$
Δ*sigB* mutant	light	$1,7 \times 10^{-4}$	$1,13 \times 10^{-5}$

Supplementary Table S5. Detailed parameters for Spectronaut (version 17) search.

```
├── DIA Analysis\Calibration
│   ├── MZ Extraction Strategy: Maximum Intensity
│   ├── Allow source specific iRT Calibration:   True
│   ├── Precision iRT:   True
│   │   ├── Exclude De-amidated Peptides:   True
│   │   └── iRT <-> RT Regression Type: Local (Non-Linear) Regression
│   ├── MS1 Mass Tolerance Strategy:   System Default
│   └── MS2 Mass Tolerance Strategy:   System Default
├── DIA Analysis\Identification
│   ├── Precursor Qvalue Cutoff:   0.001
│   ├── Precursor PEP Cutoff:   0.2
│   ├── Protein Qvalue Cutoff (Experiment):   0.01
│   ├── Protein Qvalue Cutoff (Run):   0.05
│   ├── Protein PEP Cutoff:   0.75
│   ├── Single Hit Definition:   By Stripped Sequence
│   ├── Exclude Single Hit Proteins:   False
│   ├── Exclude Duplicate Assays:   True
│   ├── Exclude Predicted Fragment Scores:   False
│   ├── Generate Decoys:   True
│   │   ├── Decoy Generation Method:   Mutated
│   │   │   └── Preferred Fragment Source:   NN Predicted Fragments
│   │   └── Decoy Limit Strategy:   Dynamic
│   │     └── Library Size Fraction:   0.1
│   └── Pvalue Estimator:   Kernel Density Estimator
├── DIA Analysis\Pipeline Mode
│   ├── Generate SNE File:   True
│   │   └── Store Ion traces in SNE:   True
│   ├── Post Analysis Reports:
│   │   ├── CV Density Line Chart:   True
│   │   ├── CVs Below X Bar Chart:   True
│   │   ├── Data Completeness Bar Chart: True
│   │   ├── Run Identifications Bar Chart: True
│   │   └── Scoring Histograms:   True
│   ├── Report Schema: C_FunGene_complex (Normal)
│   └── Reporting Unit: Across Experiment
├── DIA Analysis\Post Analysis
│   ├── Differential Abundance Testing: Paired t-test
│   │   └── Group-Wise Testing Correction:   False
│   ├── Differential Abundance Grouping:   Major Group (Quantification Settings)
│   │   └── Smallest Quantitative Unit:   Precursor Ion (Quantification Settings)
│   │     └── Use All MS-Level Quantities:   False
│   ├── Calculate Explained TIC:   None
│   ├── Calculate Sample Correlation Matrix:   True
│   └── Hierarchical Clustering: True
│     ├── Distance Metric:   Manhattan Distance
│     ├── Linkage Strategy:   Ward's Method
│     ├── Order Runs by Clustering:   True
│     └── Z-score Transformation:   False
├── DIA Analysis\Protein Inference
│   └── Protein Inference Workflow:   Automatic
│
```

```
│    └── Inference Algorithm:    IDPicker
├── DIA Analysis\PTM Workflow
│   └── PTM Localization:        True
│       ├── Probability Cutoff:  0.75
│       └── PTM Analysis:        True
│           ├── Hierarchical Clustering:     False
│           ├── Multiplicity: True
│           ├── Flanking Region:     7
│           └── PTM Consolidation:   Sum
├── DIA Analysis\Quantification
│   ├── Precursor Filtering:     Identified (Qvalue)
│   │   └── Imputation Strategy: Use Background Signal
│   ├── Proteotypicity Filter:   None
│   ├── Protein LFQ Method:      MaxLFQ
│   ├── Quantity MS Level:       MS2
│   ├── Quantity Type:   Area
│   ├── Cross-Run Normalization:         True
│   │   ├── Normalization Filter Type:   None
│   │   ├── Normalization Strategy:      Local Normalization
│   │   └── Row Selection:       Identified in at least 1 Run (Sparse)
│   ├── Interference Correction: True
│   │   ├── Only Identified Peptides:    True
│   │   ├── Exclude All Multi-Channel Interferences:     True
│   │   ├── MS1 Min:     2
│   │   └── MS2 Min:     3
│   ├── Major (Protein) Grouping:        by Protein Group Id
│   ├── Minor (Peptide) Grouping:        by Stripped Sequence
│   ├── Major Group Quantity:    Mean peptide quantity
│   ├── Major Group Top N:       True
│   │   ├── Max:         3
│   │   └── Min: 2
│   ├── Minor Group Quantity:    Sum precursor quantity
│   └── Minor Group Top N:       False
├── DIA Analysis\Workflow
│   ├── Method Evaluation:       False
│   ├── MS2 DeMultiplexing:      Automatic
│   ├── Profiling Strategy:      iRT Profiling
│   │   ├── Carry-over exact Peak Boundaries:    False
│   │   ├── Profiling Row Selection:     Minimum Qvalue Row Selection
│   │   │   └── Qvalue Threshold: 0.001
│   │   └── Profiling Target Selection:  Profile only non-identified Precursors
│   │       ├── Identification Criterion:    Qvalue
│   │       └── Threshold: 0.001
│   ├── Run Limit for directDIA Library: -1
│   └── Unify Peptide Peaks Strategy:    Select corresponding Peak
├── DIA Analysis\XIC Extraction
│   ├── XIC IM Extraction Window:        Dynamic
│   │   └── Correction Factor:   1
│   ├── XIC RT Extraction Window:        Dynamic
│   │   └── Correction Factor:   1
│   ├── MS1 Mass Tolerance Strategy:     Dynamic
│   │   └── Correction Factor:   1
│   └── MS2 Mass Tolerance Strategy:     Dynamic
│
```

```
│   └── Correction Factor:   1
├── Pulsar Search\Identification
│  ├── PSM FDR:   0.01
│  ├── Peptide FDR:   0.01
│  ├── Protein Group FDR:   0.01
│  ├── directDIA Workflow:   directDIA+ (Deep)
│  └── PTM Localization Filter: False
├── Pulsar Search\Labeling
│  └── Channels:
│     ├── Channel 1:   False
│     ├── Channel 2:   False
│     └── Channel 3:   False
├── Pulsar Search\Modifications
│  ├── Max Variable Modifications:   5
│  └── Select Modifications:
│     ├── Fixed Modifications::
│     └── Variable Modifications: :   Oxidation (M)
├── Pulsar Search\Peptides
│  ├── Enzymes / Cleavage Rules:   Trypsin/P
│  ├── Digest Type:   Specific
│  ├── Max Peptide Length:   52
│  ├── Min Peptide Length:   7
│  ├── Missed Cleavages:   2
│  └── Toggle N-terminal M:   True
├── Pulsar Search\Result Filters
│  ├── Fragment Ions:
│  │  ├── Ion AA Length:   True
│  │  │  └── N:   3
│  │  ├── Ion Charge:   False
│  │  ├── Ion Loss Type:   False
│  │  ├── Ion Type:   False
│  │  ├── m/z :   True
│  │  │  ├── Max:   1800
│  │  │  └── Min:   300
│  │  └── Relative Intensity:   True
│  │     └── Min:   5
│  └── Precursors:
│     ├── Amino Acids: False
│     ├── Best N Fragments per Peptide:  True
│     │  ├── Max:   10
│     │  └── Min:   6
│     ├── Best N Peptides per Protein Group:   False
│     ├── Channel Count:   False
│     ├── FASTA Matched:   False
│     ├── Missed Cleavage:   False
│     ├── Modifications:None
│     ├── Peptide Charge:   False
│     └── Proteotypicity:   False
├── Pulsar Search\Speed-Up
│  └── MS2 Index:   Automatic
├── Pulsar Search\Tolerances
│  └── Tolerance Parameters:
│     ├── Thermo IonTrap:
```

```
│    │    ├── Calibration Search:  Dynamic
│    │    │    ├── MS1 Correction Factor:   1
│    │    │    └── MS2 Correction Factor:   1
│    │    └── Main Search:          Dynamic
│    │         ├── MS1 Correction Factor:   1
│    │         └── MS2 Correction Factor:   1
│    ├── Thermo Orbitrap:
│    │    ├── Calibration Search:  Dynamic
│    │    │    ├── MS1 Correction Factor:   1
│    │    │    └── MS2 Correction Factor:   1
│    │    └── Main Search:          Dynamic
│    │         ├── MS1 Correction Factor:   1
│    │         └── MS2 Correction Factor:   1
│    └── TOF:
│         ├── Calibration Search:   Dynamic
│         │    ├── MS1 Correction Factor:   1
│         │    └── MS2 Correction Factor:   1
│         └── Main Search:          Dynamic
│              ├── MS1 Correction Factor:   1
│              └── MS2 Correction Factor:   1
└── Pulsar Search\Workflow
     ├── Fragment Ion Selection Strategy:   Intensity Based
     ├── In-Silico Generate Missing Channels:        False
     └── Use DNN Predicted Ion Mobility:  Auto
```

Supplementary Table S6. Detailed parameters for LC-MS/MS measurement

Reversed Phase Liquid Chromatography (RPLC)	
instrument	Ultimate 3000 RSLC (Thermo Scientific)
trap column	75 µm inner diameter, packed with 3 µm C18 particles (Acclaim PepMap100, Thermo Scientific)
analytical column	Accucore 150-C18, (Thermo Fisher Scientific)<br>25 cm x 75 µm, 2,6 µm C18 particles, 150 Å pore size
buffer system	binary buffer system consisting of 0.1% acetic acid in HPLC-grade water (solvent A) and 100% ACN in 0.1% acetic acid (solvent B)
flow rate	300 nl/min
gradient	linear gradient of buffer B from 2% up to 25%<br>(0min-2% B<br>2min-5% B<br>10min-7% B<br>70min-25% B<br>75min-40% B<br>77min-90% B<br>83min-90% B<br>85min-2% B<br>95min-2%B
gradient duration (linear gradient)	60 min (5-25%)
column oven temperature	40°C

Mass Spectrometry	
instrument	Orbitrap Exploris 480
electrospray	Nanospray Flex™
Operation mode	data-independent
MS scan resolution	120000

AGC target	3e6 (300%)
Maximum ion injection time for the MS scan	60 ms
Scan range	350 to 1200 m/z
Microscans	1
Polarity	positive
RF Lens	50%
Spectra data type	profile
Dia Properties (MS2)	
Resolution	30000
Maximum ion injection time for the MS/MS scans	auto
Normalized AGC target	3e6
Spectra data type	profile
Microscans	1
Isolation window	66
Isolation window width	13 m/z
Window overlay	2 m/z
Fixed first mass	200
HCD collision energy	30%

References

1. Nicolas P, Mäder U, Dervyn E, Rochat T, Leduc A, Pigeonneau N, Bidnenko E, Marchadier E, Hoebeke M, Aymerich S, Becher D, Bisicchia P, Botella E, Delumeau O, Doherty G, Denham EL, Fogg MJ, Fromion V, Goelzer A, Hansen A, Härtig E, Harwood CR, Homuth G, Jarmer H, Jules M, Klipp E, Le Chat L, Lecointe F, Lewis P, Liebermeister W, March A, Mars RAT, Nannapaneni P, Noone D, Pohl S, Rinn B, Rügheimer F, Sappa PK, Samson F, Schaffer M, Schwikowski B, Steil L, Stülke J, Wiegert T, Devine KM, Wilkinson AJ, van Dijl JM, Hecker M, Völker U, Bessières P, Noirot P. 2012. Condition-dependent transcriptome reveals high-level regulatory architecture in *Bacillus subtilis*. Science 335:1103–1106. doi:10.1126/science.1206848.
2. Chung YS, Dubnau D. 1998. All seven *comG* open reading frames are required for DNA binding during transformation of competent *Bacillus subtilis*. J Bacteriol 180:41–45. doi:10.1128/JB.180.1.41-45.1998.
3. BURKHOLDER PR, GILES NH. 1947. Induced biochemical mutations in *Bacillus subtilis*. Am J Bot 34:345–348.
4. Igo M, Lampe M, Ray C, Schafer W, Moran CP, Losick R. 1987. Genetic studies of a secondary RNA polymerase sigma factor in *Bacillus subtilis*. J Bacteriol 169:3464–3469. doi:10.1128/jb.169.8.3464-3469.1987.
5. Smits WK, Eschevins CC, Susanna KA, Bron S, Kuipers OP, Hamoen LW. 2005. Stripping *Bacillus*: ComK auto-stimulation is responsible for the bistable response in competence development. Molecular microbiology 56:604–614. doi:10.1111/j.1365-2958.2005.04488.x.
6. Studier FW, Moffatt BA. 1986. Use of bacteriophage T7 RNA polymerase to direct selective high-level expression of cloned genes. J Mol Biol 189:113–130. doi:10.1016/0022-2836(86)90385-2.
